# TNFα and IL-1β modify the miRNA cargo of astrocyte shed extracellular vesicles to regulate neurotrophic signaling in neurons

**DOI:** 10.1038/s41419-018-0369-4

**Published:** 2018-03-05

**Authors:** Amrita Datta Chaudhuri, Raha M. Dastgheyb, Seung-Wan Yoo, Amanda Trout, C. Conover Talbot Jr, Haiping Hao, Kenneth W. Witwer, Norman J. Haughey

**Affiliations:** 1 Department of Neurology, Richard T Johnson Division of Neuroimmunology and Neurological Infections, Johns Hopkins University School of Medicine, Baltimore, MD USA; 2 Institute of Basic Biomedical Sciences, Johns Hopkins University School of Medicine, Baltimore, MD USA; 3 Molecular and Comparative Pathobiology, Johns Hopkins University School of Medicine, Baltimore, MD USA

## Abstract

Astrocytes are known to be critical regulators of neuronal function. However, relatively few mediators of astrocyte to neuron communication have been identified. Recent advancements in the biology of extracellular vesicles have begun to implicate astrocyte derived extracellular vesicles (ADEV) as mediators of astrocyte to neuron communication, suggesting that alterations in the release and/or composition of ADEVs could influence gliotransmission. TNFα and IL-1β are key mediators of glial activation and neuronal damage, but the effects of these cytokines on the release or molecular composition of ADEVs is unknown. We found that ADEVs released in response to IL-1β (ADEV-IL-1β) and TNFα (ADEV-TNFα) were enriched with miRNAs that target proteins involved in neurotrophin signaling. We confirmed that miR-125a-5p and miR-16-5p (both enriched in ADEV-IL-1β and ADEV-TNFα) targeted NTKR3 and its downstream effector Bcl2. Downregulation of these targets in neurons was associated with reductions in dendritic growth, dendritic complexity, reduced spike rates, and burst activity. Molecular interference of miR-125a-5p and miR-16-5p prevented ADEV-IL-1β from reducing dendritic complexity, spike, and burst rates. These findings suggest that astrocytes respond to inflammatory challenge by modifying the miRNA cargo of ADEVs to diminish the activity of target neurons by regulating the translational expression of proteins controlling programs essential for synaptic stability and neuronal excitability.

## Introduction

Astrocytes occupy a unique niche in the CNS that allows them to regulate neuronal activity through interactions with pre- and post-synaptic specifications. This strategic location of astrocytes allows them to coordinate, regulate, and adapt the central nervous system to physiological demands, such as those required for learning, sensory experiences, response to infection or disease^1^. Astrocytes accomplish these functions through the selective uptake of synaptic transmitters and peptides, and through the regulated release of a neuromodulatory compounds that include neurotrophic factors, neurotransmitters, hormones, peptides, lipid products, energy metabolites, cytokines, and growth factors^2^. This regulated release occurs through several different mechanisms that include exocytosis, diffusion, active, and passive transport^2^. A recent addition to the mechanisms for astroglial communication is through the release of secretory vesicles. Astrocytes contain multiple populations of secretory vesicles that include exosomes, microvesicles, synaptic-like microvesicles, dense-core granules, and lysosomes that each contains a complex cargo of lipids, proteins, RNA, and miRNA that can regulate the activity of target cells^3, 4^. Recent advancements in the biology of extracellular vesicles (EVs) have begun to implicate astrocyte derived extracellular vesicles (ADEVs) as mediators of glia to neuron communication^5, 6^, suggesting that alterations in the release and/or cargo of ADEVs could differentially modulate neuronal function.

ADEVs are constitutively shed from astrocytes, and the rate of ADEV release can be augmented or suppressed by stimulation^7^. Constitutively shed ADEVs have been reported to have neurotrophic and protective roles^8^, and ADEVs secreted in response to ischemic, oxidative, nutrient deprivation, or thermal stress carry hsp70 and synapsin-I, or leukemia inhibitory factor that promote neuronal survival^6, 9, 10^. In contrast, ADEVs may also promote neurodegeneration by spreading pathology in disease conditions^11–19^. Disease-specific cargo of EVs include the prion protein in spongiform encephalopathy^11^, tau and amyloid-β1-42 (Aβ1-42), ceramide and PAR4 in models of Alzheimer’s Disease (AD)^12–16^, α-synuclein and LRRK2 in rodent models of Parkinson’s disease^17, 18^ superoxide dismutase1 (SOD1) in rodent models of Atrophic Lateral Sclerosis^19^, and neurotoxic HIV-1 proteins in cell models of infection and neurodegenerative disease^20, 21^. Inhibiting EV release appears to prevent the spreading of Tauopathy^12^. These data suggest that the protein cargo of ADEVs can be modified by stimulation, and the presence of disease.

The protected RNase-free environment of EVs provides a particular advantage for the intercellular transport of microRNAs (miRNA). It is not currently known if there is a stimulus-dependent inclusion of particular miRNA cargo in ADEVs under physiological or disease conditions. Likewise, very little is currently known regarding the effects of particular ADEV miRNAs on neuronal function. ADEVs shed in response to lipopolysaccharide-induced stress contain miR-34a that sensitizes neurons to toxic insults by downregulating the anti-apoptotic protein BCl2 (ref. ^22^), and exposure of astrocytes to morphine and the HIV protein Tat increases the release of miR-29b in ADEVs that downregulates neuronal PDGF-B expression and viability in target neurons^23^.

In this study, we identify miRNAs enriched in ADEVs shed in response to ATP (ADEV-ATP), TNFα (ADEV-TNFα), and IL-1β (ADEV-IL-1β) compared with the miRNA cargo of constitutively released ADEVs (ADEV-CR). We further identify two miRNAs enriched in both ADEV-TNFα and ADEV-IL1β that downregulate the expression of neurotrophin receptor NTRK3 (TRKC), and its downstream effector Bcl2. The resulting simplification of dendritic complexity dampens neural network activity, but does not modify neural network connectivity.

## Results

### ADEVs regulate dendritic growth and complexity

We first determined if the stimulus used to evoke ADEV shedding modified the number and size of ADEVs released from primary astrocytes. In fresh FBS-free media, we exposed astrocytes for 2 h to ATP (10 μM), IL-1β or TNFα (200 ng/mL each) and compared the size and quantity of exosomes released with no-treatment (trophic factor withdrawal; TFW) using nanoparticle tracking analysis. Astrocytes shed 6.7 ± 0.17 × 10^9^ ADEVs/mL over a 2 h time frame in response to TFW. Stimulation with ATP increased the number of EVs shed to 8.8 ± 0.51 × 10^9^ ADEVs/mL, IL-1β to 9.1 ± 0.23 × 10^9^ ADEVs/mL, and TNFα to 8.3 ± 0.56 × 10^9^ ADEVs/mL. The size of ADEVs shed from astrocytes in response to TFW was 105.93 ± 2.7 nm, and size was reduced to 86.03 ± 4.9 nm by stimulation with ATP. IL-1β or TNFα did not significantly modify the size of ADEVs (Supplementary Table 1).

**Fig. 1:**
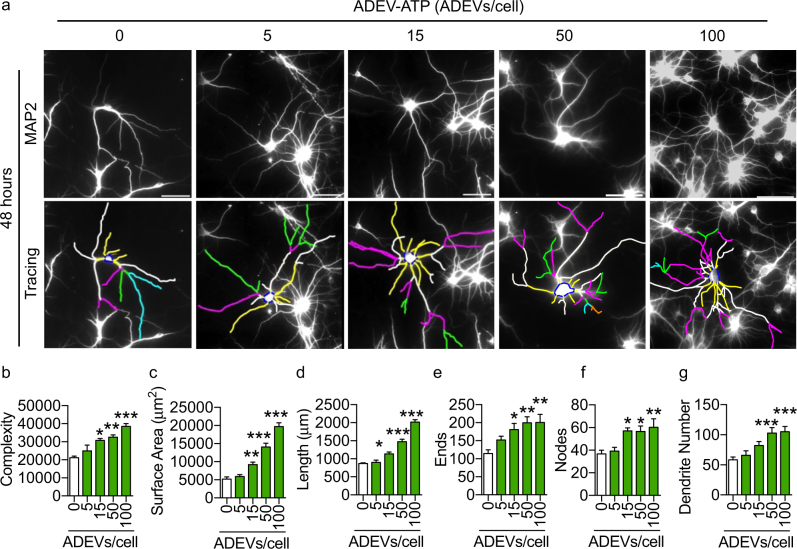
ADEV-ATP enhances dendritic arborization. **a** Representative fluorescent images of MAP2 immunopositive hippocampal neurons (top panels) and Neurolucida dendrite tracings (bottom panels) following a 48 h exposure to the indicated concentrations of ADEV-ATP. Quantitative data show **b** dendritic complexity, **c** surface area, **d** dendritic length, **e** number of ends, **f** nodes, and **g** total dendrite number for the indicated concentrations of ADEV-ATP. Data are mean ± SEM of 15–20 neurons from three independent experiments. One-way ANOVA with Tukey’s post hoc comparisons. *p < 0.05, **p < 0.01, ***p < 0.001 compared to control

**Fig. 2:**
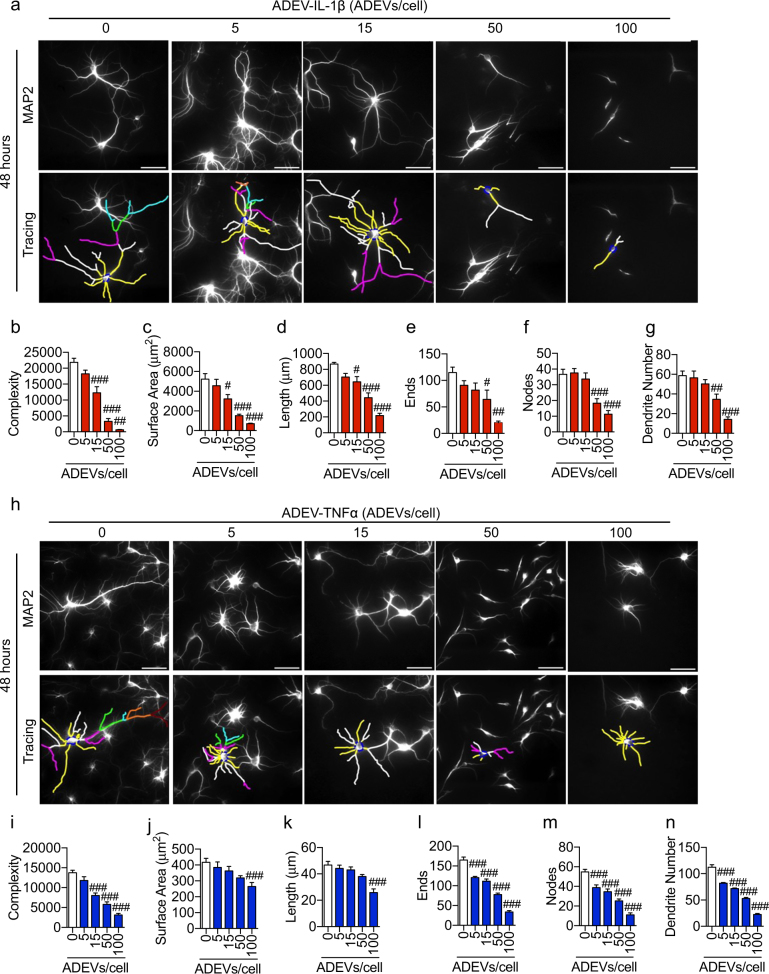
ADEV-IL-1β and ADEV-TNFα dose-dependently reduce dendritic complexity. **a** Representative fluorescent images of MAP2 immunopositive hippocampal neurons (top panels) and Neurolucida dendrite tracings (bottom panels) following a 48 h exposure to the indicated concentrations of ADEV-IL-1β. Quantitative data show (**b**) dendritic complexity, **c** surface area, **d** dendritic length, **e** number of ends, **f** nodes, and **g** total dendrite number for the indicated concentrations of ADEV-IL-1β. **h** Representative fluorescent images of MAP2 immunopositive hippocampal neurons (top panels) and neurite tracings (bottom panels) following a 48 h exposure to the indicated concentrations of ADEV-IL-1β. Quantitative data show **i** dendritic complexity, **j** surface area, **k** dendritic length, **l** number of ends, **m** nodes, and **n** total dendrite number for the indicated concentrations of ADEV-IL-1β. Data are mean ± SEM of 15–20 neurons from three independent experiments. One-way ANOVA followed by Tukey’s post hoc comparisons. ^#^*p* < 0.05, ^##^*p* < 0.01, ^###^*p* < 0.001 compared to control

We next exposed primary hippocampal neurons to ADEV-ATP, ADEV-IL-1β, or ADEV-TNFα. In mature neurons (DIV 14–21) ADEV-ATP produced a dose-dependent increase in neurite length, dendritic complexity, number of nodes, number of dendrites, ends, and total surface area (Fig. 1a–g). Peak trophic effects were observed 48 h following exposure with a minimum effective dose of 15 ADEVs per neuron (Fig. 1a–g). We observed a similar trophic effect when developing (DIV 3) hippocampal neurons were exposed to ADEV-ATP (Supplementary Figure 1). Mature hippocampal neurons treated with ADEV-IL-β showed a dose dependent reduction in neurite length, dendritic complexity, number of nodes, number of dendrites, ends, and total surface area, with a lowest effective dose of 50 ADEVs per neuron (Fig. 2a–g). Exposing neurons to ADEV-TNFα resulted in similar reductions of neurite length, dendritic complexity, number of nodes, number of dendrites, ends, and total surface area (Fig. 2h–n). An inhibition of neurite development was observed when DIV 3 hippocampal neurons were exposed to ADEV-IL-1β (Supplementary Figure 2).

To confirm that the decrease in dendritic outgrowth and complexity was not produced by IL-1β or TNFα contamination in our EV preparations, we first pretreated neurons for 30 min with an IL-1 receptor antagonist (IL-1RA, 20 ng/mL), or an inhibitor of TNFα activity (SPD304, 50 μM), then exposed the neurons to 50 ADEV-IL-1β/neuron or ADEV-TNFα/neuron for 48 h before imaging dendrites. Under these conditions, we found reductions in neurite length, dendritic complexity, number of nodes, number of dendrites, ends, and total surface area (Supplementary Figure 3) that were similar in magnitude to data from neurons exposed to 50 ADEV-IL-1β/neuron or ADEV-TNFα/neuron alone without any pretreatment (Fig. 2). These data demonstrate that reductions in dendritic complexity induced by ADEV-IL-1β or ADEV-TNFα are not mediated by IL-1β or TNFα contamination in our EV preparations.

### ADEV-IL-1β and ADEV-TNFα are enriched with miRNA targeting the neurotrophin pathway

**Fig. 3:**
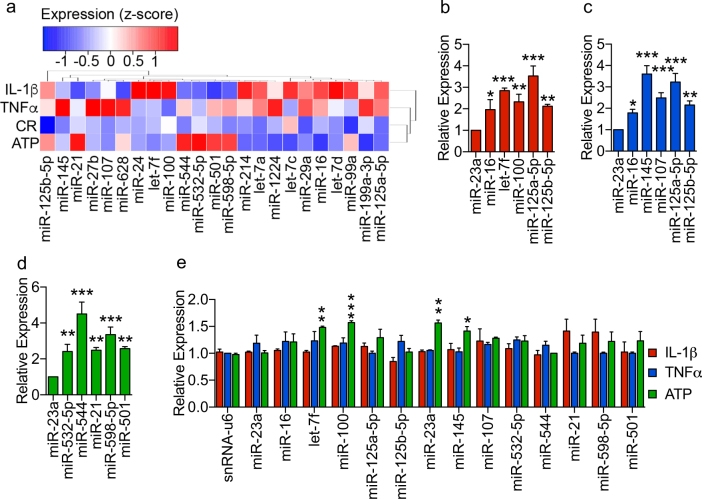
Stimulus-depended enrichment of miRNA cargo in ADEVs. **a** Heatmap and hierarchical clustering showing relative levels for microRNAs enriched >1.5-fold in ADEVs shed in response to the indicated treatment conditions compared with ADEV-CR. **b** qRT-PCR validation of the miRNAs most enriched in ADEV-IL-1β. **c** qRT-PCR validation of the miRNAs most enriched in ADEV-TNFα. **d** qRT-PCR of the microRNAs most enriched in astrocyte ADEV-ATP. **e** qRT-PCR showing levels of the indicated miRNAs in cultured astrocytes following treatment with ATP (10 μM), IL-1β or TNFα (200 ng/ml) for 2 h. Data are mean ± SEM of *n* = 3 independent experiments. One-way ANOVA with Tukey’s post hoc comparisons. *p < 0.05, **p < 0.01, ***p < 0.001 compared with CR

We next sought to determine whether the miRNA cargo of ADEVs was modified by the stimulus used to provoke EV release. We detected a total of 131 distinct miRNAs in ADEVs (Supplementary Table 2). Compared with ADEV-CR, 7 miRNAs were enriched in ADEV-ATP, 10 in ADEV-IL-1β, and 15 in ADEV-TNFα (Fig. 3a, and Supplementary Table 2). The top five enriched miRNAs in each treatment condition were validated by qRT-PCR (Fig. 3b–d). All the validated miRNAs in each treatment condition were highly expressed in astrocytes, but cellular levels of these miRNAs in astrocytes were not noticeably reduced following IL-1β or TNFα stimulation (Fig. 3e). Stimulation with ATP resulted in significant increase in cellular levels of let-7f, miR-100, miR-23a, and miR-145.

**Fig. 4:**
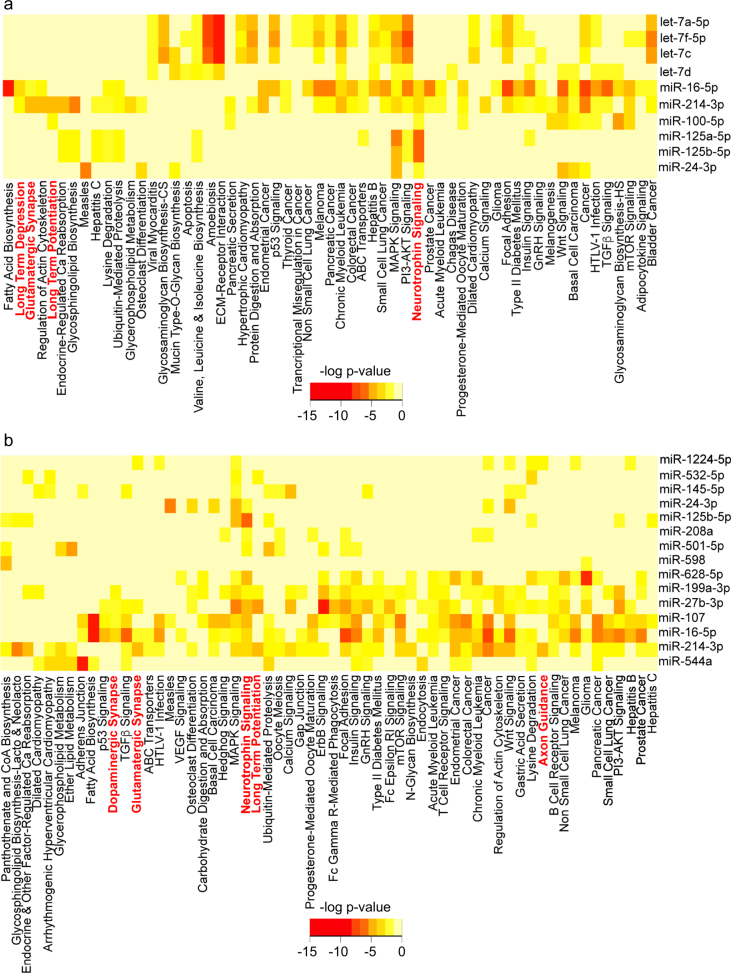
Bioinformatic interrogation of miRNAs enriched in ADEV-IL-1β and ADEV-TNFα identified several neuronal-specific pathways. **a** Pathway analysis of predicted miRNA targets enriched in ADEV-IL-1β using DIANA miRPATH. **b** Pathway analysis of predicted miRNA targets enriched in ADEV-TNFα using DIANA miRPATH. Heat maps depict levels of miRNA enrichment for the indicated signaling pathways. Darker colors (orange to red) indicate more enrichment of predicted microRNA targets belonging to the corresponding pathway. Several predicted pathways were associated with neurite outgrowth, dopamine and glutamatergic synapses, long-term potentiation, long-term depression, and neurotrophin signaling (shown in red)

**Fig. 5:**
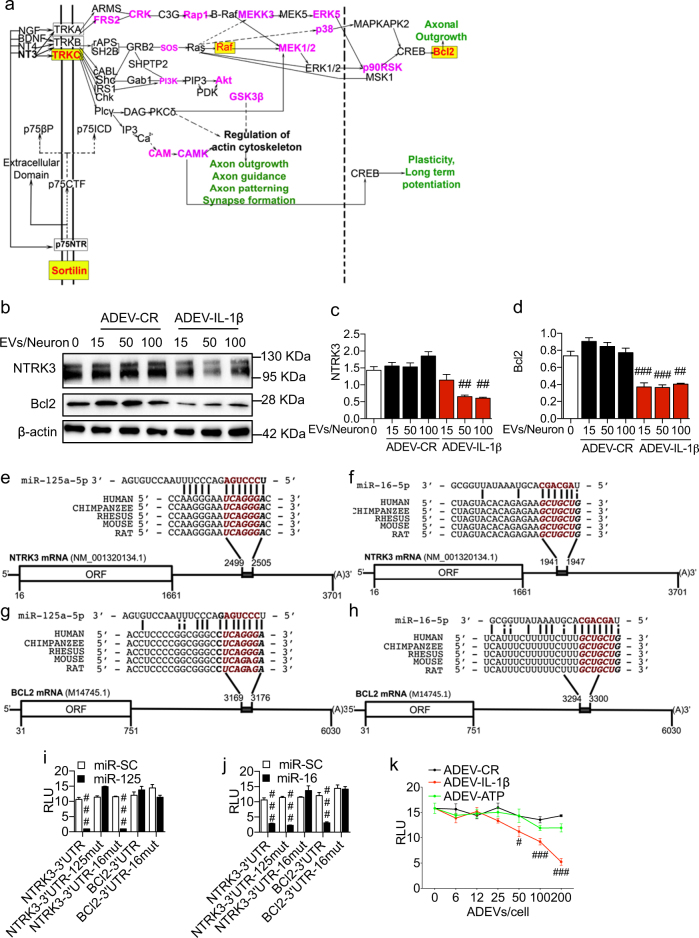
MiR-125a-5p and miR-16-5p target NTRK3 and Bcl2. **a** Schematic diagram of the neurotrophin-signaling pathway. The mRNAs in red text and yellow highlight are predicted targets of at least two miRNAs (miR-125a-5p and miR-16-5p) enriched in ADEV-TNFα, or ADEV-IL-1β. The mRNAs in magenta text are predicted targets of 1 miRNA enriched in ADEV-TNFα, or ADEV-IL-1β. **b** Representative western blot for NTRK3 and Bcl2 in primary neurons treated with ADEV-CR or ADEV-IL-1β at the indicated particle dose. Densitometry quantitation of (**c**) NTRK3, and (**d**) Bcl2 levels normalized to β-actin. Schematic diagrams show (**e**) miR-125a-5p binding site on NTRK3 3′-UTR, **f** miR-125a-5p binding site on BCL2 3′-UTR, **g** miR-16-5p binding site on NTRK3 3’UTR, and **h** miR-16-5p binding site on BCL2 3′-UTR. **i** 3′-UTR luciferase reporter assay showing functional binding of miR-125a-5p to the 3′-UTR of NTRK3 and Bcl2. **j** 3′-UTR luciferase reporter assay showing functional binding of miR-16-5p to the 3′-UTR of NTRK3 and Bcl2. **k** 3′-UTR luciferase reporter assay in cells transfected with NTRK3 3′-UTR followed by treatment with ADEV-CR, ADEV-ATP or ADEV-IL-1β at the indicated ADEV dose. Data are mean ± SEM of a minimum *n* = 3 independent experiments per condition. One-way ANOVA with Tukey’s post hoc comparisons. ^#^*p* < 0.05, ^##^*p* < 0.01, ^###^*p* < 0.001 compared to control

We next used DIANA miRPATH^24^ to identify putative signaling pathways modified by ADEV-TNFα or ADEV-IL-1β based on predicted miRNA targets. Pathways identified included the neurotrophin signaling pathway, axon guidance, long-term potentiation, long-term depression, glutamatergic synapse, and dopaminergic synapse (Fig. 4). Fourteen genes in the neurotrophin signaling pathway were predicted to be targeted by at least one microRNA enriched in ADEV-IL-1β or ADEV-TNFα, and two key genes in this pathway (NTRK3/TRKC and BCl2) were predicted to be targeted by both miR-125a-5p and miR-16-5p that were enriched in ADEV-IL-1β and ADEV-TNFα (Fig. 5a). Similar analyses using the miRNA content of ADEV-ATP did not identify cellular targets associated with neurite outgrowth or plasticity (Supplemental Fig. 3). NTRK3/TRKC is required for dendritic growth and branching^25^, and Bcl2 is known to regulate neurite outgrowth^26^. Western blot analysis confirmed downregulation of NTRK3 and Bcl2 in neurons treated with ADEV-IL-1β (Fig. 5b–d). These findings suggest that ADEV-IL-1β and ADEV-TNFα may reduce neurite complexity by downregulating neurotrophin signaling, and the cellular capacity to maintain dendritic growth.

Bioinformatic analysis identified conserved binding sites for miR-125a-5p and miR-16-5p on the 3′-UTR of NTRK3 mRNA (Fig. 5e, f). The 3′-UTR of Bcl2 mRNA contained a single-conserved binding site for miR-16-5p (Fig. 5g, h). We validated binding sites on the 3′-UTR of NTRK3 and Bcl2 mRNA for miR-125a-5p and miR-16-5p by cloning the 3′-UTRs of NTRK3 and Bcl2 into the pmiRGLO plasmid downstream of the firefly luciferase gene. Luciferase reporter plasmids were cotransfected into HEK293T cells along with miRNA mimics for miR-125a-5p and miR-16-5p, or a scrambled microRNA mimic (miR-SC) as a control. Transfection of HEK293T cells with the miR-125a-5p mimic produced a dramatic decrease of luciferase expression from the NTRK3-3′-UTR plasmid that was abolished by mutating of two nucleotides in the miR-125a-5p binding site of the NTRK3-3′-UTR (Fig. 5i). Mutation of two nucleotides in the miR-16-5p binding site of the NTRK3-3′-UTR did not affect luciferase expression in cells transfected with the miR-125a-5p mimic (Fig. 5i), and the miR-125a-5p mimic did not decrease luciferase activity in cells transfected with the Bcl2-3′-UTR plasmid, consistent with the lack of a conserved binding site for miR-125a-5p on the Bcl2-3′-UTR (Fig. 5i). The miR-16-5p mimic decreased luciferase expression from both the NTRK3-3′-UTR plasmid, and the Bcl2-3′-UTR plasmid (Fig. 5j), consistent with conserved binding sites for miR-16-5p in both the NTRK3-3 and Bcl2-3′-UTRs. Mutating two nucleotides in the miR-16-5p binding site on the NTRK3-3′-UTR or the Bcl2-3′-UTR abolished luciferase expression (Fig. 5j). Exposing HEK293T cells transfected with the NTRK3-3′-UTR luciferase reporter plasmid with ADEV-IL-1β for 24 h produce a dose dependent decrease in luciferase expression that was not apparent in similar cells exposed to ADEV-ATP (Fig. 5k). These data confirm that miR-125a-5p, and miR-16-5p enriched in ADEV-IL-1β both target NTRK3 mRNA, and miR-16-5p selectively targets Bcl2 mRNA.

### miR-125a-5p and miR-16-5p in ADEV-IL-1β reduce neuronal expression of NTRK3 and Bcl2

**Fig. 6:**
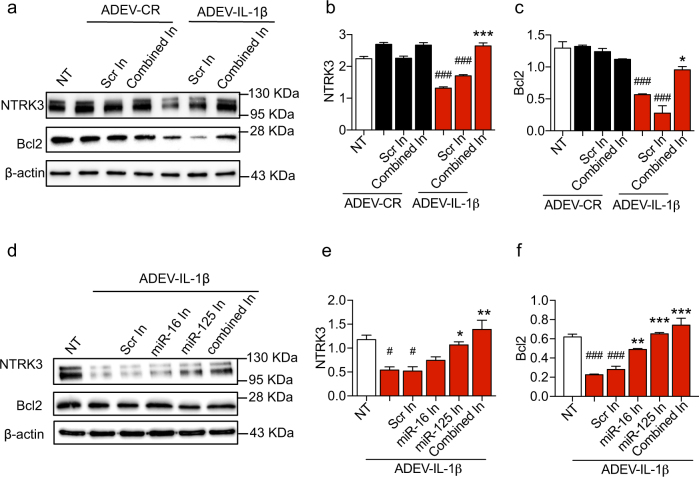
Molecular interference of miR-125a-5p and miR-16-5p prevents ADEV-IL-1β from reducing NTRK3 and BCl2 expression. **a** Representative western blot showing NTKR3 and Bcl2 protein expression in neurons 24 h following treatment with ADEV-CR or ADEV-IL-1β alone or in combination with antisense oligonucleotide inhibitors for miR-125 and miR-16 (20 pmole each; Combined In). Scrambled oligonucleotide inhibitors were used as a control (Scr In). Quantitative densitometry analysis of (**b**) NTRK3, and (**c**) Bcl2 for the indicated treatment conditions. **d** Representative western blot showing NTKR3 and Bcl2 protein expression in neurons 24 h following treatment with ADEV-CR or ADEV-IL-1β alone or in combination with antisense oligonucleotide inhibitors either miR-1 25 or miR-16 alone or in combination (20 pmole each; Combined In). Scrambled oligonucleotide inhibitors were used as a control (Scr In). Quantitative densitometry analysis of (**e**) NTRK3, and (**f**) Bcl2 for the indicated treatment conditions. Data are mean ± SEM of *n* = 3 independent experiments. One-way ANOVA with Tukey’s post hoc test. *p < 0.05, **p < 0.01, ***p < 0.001 increased compared to no treatment (NT), ^#^*p* < 0.05, ^###^*p* < 0.001 decreased compared with NT

As ADEV-IL-β and ADEV-TNFα were both enriched with miR-125a-5p and miR-16-5p, all further experiments were conducted with ADEV-IL-1β. We transfected ADEV-IL-1β with antisense oligonucleotide inhibitors for miR-125a-5p and miR-16-5p to confirm that these miRNAs were responsible for the observed decrease in neuronal NTRK3 and Bcl2 expression. Exposure of neurons to ADEV-IL-1β pre-transfected with antisense oligonucleotide inhibitors for miR-125a-5p and miR-16-5p prevented decreases of NTRK3 and Bcl2 protein expression. Transfection of ADEV-IL-1β with a scrambled antisense oligonucleotide inhibitor did not prevent decreases in neuronal NTRK3 and Bcl2 protein expression (Fig. 6a–c). To determine the relative contribution of miR-125a-5p and miR-16-5p, we exposed neurons to ADEV-IL-1β pre-transfected with each antisense inhibitor separately. Consistent with our 3′-UTR luciferase reporter assay results, we found that molecular interference with either miR-125a-5p or miR-16-5p prevented ADEV-IL-1β from reducing neuronal NTRK3 expression. Reductions in Bcl2 expression following exposure to ADEV-IL-1β were selectively rescued by molecular interference with miR-16-5p, but not by interference with miR-125a-5p (Fig. 6d–f).

### miR-125a-5p and miR-16-5p regulate dendritic complexity

**Fig. 7:**
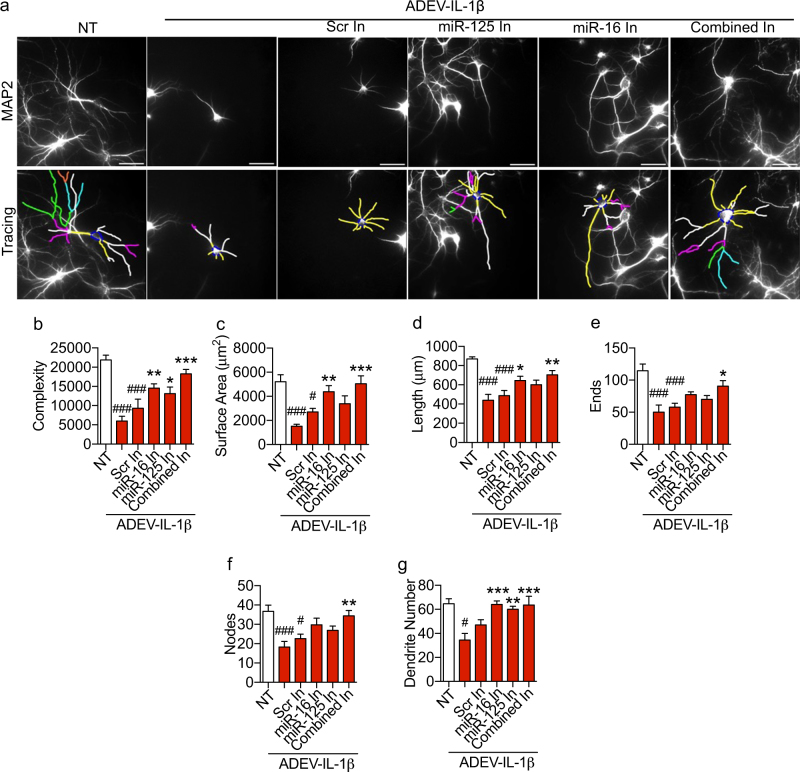
Molecular interference of miR-125a-5p and miR-16-5p prevents ADEV-IL-1β from reducing dendritic arborization. **a** Representative fluorescent images of MAP2 immunopositive hippocampal neurons (top panels) and Neurolucida dendrite tracings (bottom panels) following a 48 h exposure to the indicated treatment conditions. Quantitative data show (**b**) dendritic complexity, **c** surface area, **d** dendritic length, **e** number of ends, **f** nodes, and **g** total dendrite number for the indicated treatment conditions. ADEV-IL-1β particle dose was 50 ADEVs/cell, and antisense oligonucleotide inhibitors for miR-125 and miR-16 were used at 20 pmole. Data are mean ± SEM for the indicated treatment conditions. One-way ANOVA followed by Tukey’s post hoc test. *p < 0.05, **p < 0.01, ***p < 0.001 increased compared to NT. ^#^*p* < 0.05, ^##^*p* < 0.01, ^###^*p* < 0.001 decreased compared to NT

Reductions in neurite complexity, surface area, length, and dendritic number were prevented by pre-transfecting ADEV-IL-1β with antisense oligonucleotide inhibitors targeted to miR-125a-5p (Fig. 7a–d, g). Reductions in dendritic complexity, and number were prevented by pre-transfecting ADEV-IL-1β with antisense oligonucleotide inhibitors targeted to miR-16-5p (Fig. 7a, b, g). Transfection of ADEV-IL-1β with oligonucleotide inhibitors targeted to both miR-125a-5p and miR-16-5p rescued all measures of dendritic morphology, including neurite complexity, surface area, length, ends, nodes, and dendritic number (Fig. 7a–g). Transfecting ADEV-IL-1β with scrambled oligonucleotides did not prevent simplifications in dendritic morphology (Fig. 7a–g).

We confirmed that miR-125a-5p and miR-16-5p carried in ADEV-IL-1β mediated the observed reductions in dendritic complexity using DOTAP to encapsulate and directly deliver miR-125a-5p and miR-16-5p to neurons. DOTAP-encapsulated miR-SC was used as control. Direct delivery of miR-125a-5p and miR-16-5p reduced neurite length, dendritic complexity, number of nodes, number of dendrites, ends, and total surface area. Delivery of DOTAP-encapsulated miR-SC did not affect any parameter of dendritic complexity measured (Supplementary Figure 5)

### ADEV-IL-1β dampened neural network activity

**Fig. 8:**
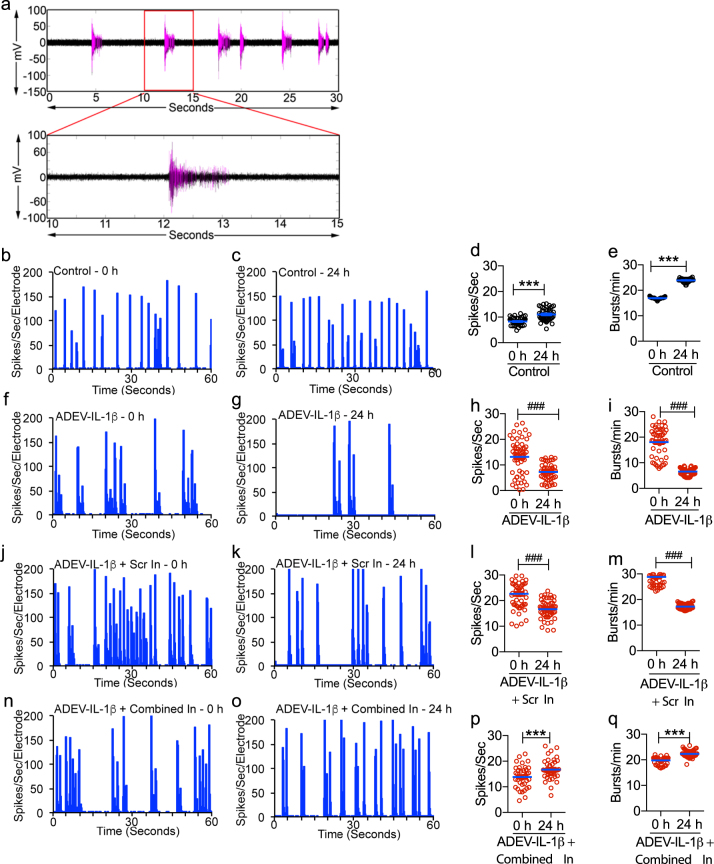
ADEV-IL-1β reduces neuronal activity but not connectivity. **a** Representative recording of population spikes (each purple vertical line), and bursts (purple clusters with >4 spikes/s) from an individual electrode of a multichannel electrode array. Representative tracings show averages of spikes/second/electrode, and associated scatter plots show quantitation of spike and burst rates for (**b**–**e**) Control, (**f**–**i**) ADEV-IL-1β (particle dose of 50 ADEVs/cell), (**j**–**m**) ADEV-IL-1β+ Scrambled oligonucleotide (Scr In, 20 pmole), and (**n**–**q**) ADEV-IL-1β+ oligonucleotide inhibitors for miR-125 and miR-16 (Combined In, 20 pmole each). Data are mean ± SEM. Paired *t*-tests were performed to compare spike and burst rate of each electrode before and after treatment. ***p < 0.001 increased compared to baseline and ^###^*p* < 0.001 decreased compared to baseline

We monitored neuronal spike and burst activity beginning at DIV 14 in primary hippocampal neurons cultured on multichannel electrode arrays (Fig. 8a). When neurons exhibited mature spontaneous spike and burst activity (21–26 DIV) they were exposed to ADEV-IL-1β, or ADEV-IL-1β pre-transfected with oligonucleotide inhibitors targeted to miR-125a-5p and miR-16-5p. Neuronal activity was measured at baseline (before exposure to ADEVs), and 24 h following treatment with ADEVs. We normalized spike and burst rates to pre-treatment activity to control for batch differences in baseline activity. Qualitative characterization of the firing activity of neurons around individual electrodes in the culture plates was summarized in binned raster plots, which shows a temporal sequence of spikes binned into 0.2 s intervals. In control cultures, neurons increased spike activity by 132.9 ± 5.3% of baseline, and burst activity by 140.9 ± 1.08% of baseline activity over the 24 h time frame (Fig. 8b–e). In contrast, neurons exposed to ADEV-IL-1β decreased spike activity by 54.85 ± 3.28% of baseline, and burst activity by 37.41 ± 1.02% of baseline (Fig. 8f–i). Exposure of astrocytes to ADEV-IL-1β pre-transfected with oligonucleotide inhibitors targeted to miR-125a-5p and miR-16-5p prevented reductions in neuronal activity (111.1 ± 4.74% increase in the mean spike rate, and 91.72 ± 1.03% increase in the mean burst rate of above baseline) (Fig. 8j–m). Pre-treatment of ADEV-IL-1β with a scrambled oligonucleotide inhibitor did not prevent reductions in the spike (73.32 ± 2.49% reduction from baseline), or burst rate (59.86 ± 5.42% reduction from baseline) (Fig. 8n–q). ADEV-IL-1β did not modify neural network connectivity within 24 h of exposure (Supplementary Figure 4).

## Discussion

Astrocytes (and other cells) constitutively release extracellular vesicles. While some of this release appears to be a form of cellular trash removal^5^, there is considerable evidence that these constitutively released vesicles are a mechanism for the homeostatic regulation of neuronal function^8^. We found ADEV-CR was enriched with miR-29b, miR-29c, miR-130a, miR-154, and miR-466d compared to ADEV-ATP, ADEV-TNFα, and ADEV-IL-1β. Consistent with a role in the homeostatic regulation of neuronal function miR-29b has been reported to target HDAC4, a histone deacetylase that is abundantly expressed in the brain and essential for synaptic transmission, information processing, long-term memory^27^. HDAC4 is normally localized in the cytoplasm, and nuclear translocation of HDAC4 results in suppression of genes related to synaptic function, such as CamKIIα, Syn2, Homer1, Homer2, Vglut1, Snap25, Dlg2, Rab3c, Lgi1, and FGF13 (ref. ^27^). MiR-29c is expressed during early stages of brain development and promotes neurite outgrowth by decreasing PTEN expression^28^, and by positively regulating the expression of BDNF by enhancing activity of the extracellular signal-regulated kinase pathway^29^. However, there is also a single report suggesting that miR-29c may be a negative regulator of axonal growth under conditions of hypoglycemic stress^30^. MiR-130a has been reported to target ROCK1, a downstream effector of RhoA-GTPase^31^, that induces neurite retraction^32^. Inhibition of ROCK1 has been demonstrated to protect neurons in models of spinal cord-injury, promotes dendritic spine maturation, improves spatial learning, and working memory in rodents^33^. MiR-154 is known to inhibit the expression of Dickkopf-related protein 2, with a resultant upregulation in β-catenin expression and activation of the classical Wnt signaling pathway^34^. Wnt signaling is critical for the maintenance of synaptic structures through the promotion of dendritic spine formation and maintenance^35^, and promotes neuronal survival through the regulation of neurotrophin expression^36^. Together these data suggest that the constitutive release of EVs from astrocytes may participate in the homeostatic maintenance of neuronal synapses through the regulation of transcriptional programs essential for synaptic transmission, and information processing. This homeostatic delivery of ADEV cargo to neurons may be perturbed when the cargo of ADEVs is modified by inflammatory cytokines, or other stimuli.

Cytokines regulate a variety of functions in the central nervous system. IL-1β and TNFα play important roles in regulating neuronal development, neuronal excitability, sleep, and neuroendocrine functions^37, 38^. The expression of inflammatory cytokines is upregulated ~1000-fold in response to infection or damage, and even a peripheral inflammatory cytokine response can signal to the central nervous system via the circumventricular organs that lack a blood brain barrier, or through interactions with vascular endothelium^39^. Signaling into the brain via these routes evokes a response from perivascular astrocytes that we propose includes an increased release of ADEVs with modified cargo. We found that IL-1β and TNFα increased the release of ADEVs ~1.5-fold, likely due to the direct linkage of the IL-1 and TNF receptors to the sphingomyelin hydrolase nSMase2 through the protein linkers RAC-1 and FAN^40, 41^. NSMase2 promotes the formation and budding of EVs^42^. The mechanisms for differential enrichment of particular miRNAs into ADEVs following ATP, TNFα, or IL-1β are less clear. Previous studies have reported differential sorting of miRNA into EVs^43^, and several mechanisms for miRNA loading into EVs including lipid-mediated RNA loading, RNA-binding protein-assisted RNA loading, loading by non-templated 3′-end uridylation of microRNAs^44–46^. However, mechanisms regulating stimulus-dependent inclusion/exclusion of particular miRNA cargo are currently unknown.

We found that ADEV-IL-1β was enriched in let-7a, let-7c, let-7d, let-7f, miR-16, miR-214, miR-100, miR-125a-5p, miR-125b-5p, and miR-24, while ADEV-TNFα was enriched with miR-27b, miR-145, miR-107, miR-628, miR-544, miR-598-5p, miR-16, miR-1224, miR-214, miR-199a-3p, miR-501, miR-125b-5p, miR-24, miR-532-5p, and miR-208a. The majority of these miRNAs target proteins required to support neurotrophic and synaptic functions. For example, several of these miRNAs target proteins regulating neurogenesis, including the Kirsten ras oncogene homolog, Forkhead box protein (Let-7a^47, 48^), the nuclear receptor TLX (homolog of the Drosophila tailless gene) (Let-7d^49^), Sox-2 and Sox-9 (MiR-145 (ref. ^50, 51^)). Other miRNAs target proteins regulating neuronal survival and synaptogenesis, including the insulin-like growth factor-1 receptor (IGF-1R), p21-activated kinase 1, (Let-7c^52, 53^), the RNA binding protein insulin like growth factor 2 binding protein 1 (let-7f^54^), the mechanistic target of rapamycin (miR-100 (ref. ^55^)), and glutamate transporter 1 (miR-107 (ref. ^56^)). Others target anti-inflammatory and anti-apoptotic functions including mitogen-activated protein kinase phosphatase 1 (Let-7a^57^), heat shock protein (MiR-16 (ref. ^58^)). A smaller number of these miRNAs target pro-apoptotic proteins, such as apoptosis protease activating factor-1 (MiR-27b^59^), and repressors of dendritic/synaptic growth including the polycomb group protein Bmi1 (MIR-27b^60^), and the Schizophrenia-associated gene Quaking (MiR-214 (ref. ^61^)).

A bioinformatic analyses of the miRNAs enriched in ADEV-TNFα and ADEV-IL-1β identified multiple targets in the neurotrophin signaling pathway as potential cellular effectors for reductions in dendritic arborization observed following treatment of neurons with ADEV-TNFα and ADEV-IL-1β. We identified and validated miR-125a-5p and miR-16-5p (enriched in ADEV-IL-1β and ADEV-TNFα) as miRNAs that target NTRK3 and Bcl2 in neurons. NTRK3 (also known as TrkC) is a transmembrane tyrosine kinase receptor for neurotrophin 3 (NT-3). Binding of NT-3 to NTRK3 results in receptor dimerization, phosphorylation, activation of downstream kinases and signaling molecules that ultimately promote neuronal survival, differentiation and neurite outgrowth^62, 63^. Additionally, NTRK3 can stimulate the Ras-Map/Erk signaling cascade to phosphorylate and activate the transcription factor CREB^64^ that regulates the expression of genes supporting neuronal survival and differentiation^65^. Activation of CREB by NTRK3 has been associated with the induced expression of Bcl2 (ref. ^66^), axon regeneration and neurite outgrowth both in vitro and in vivo^26, 67^. These data suggest that inflammatory cytokines promote the release of ADEVs enriched with miRNAs that downregulate NTRK3 and Bcl2. A reduced expression of these neuronal targets slowed dendritic growth in developing neurons, simplified dendritic complexity in mature neurons, and reduced neuronal excitability. We speculate that astrocytes respond to inflammatory cytokine stimulation by packaging and releasing EVs enriched with miRNA cargo that dampen the excitability of neurons, and suggest that this may be a protective response to brain inflammation. These conclusions are consistent with findings from other studies that found ADEVs shed in response to cellular stressors, including nutrient deprivation, oxidative stress, ischemic, and thermal stress carry a variety of neuroprotective proteins that include hsp70, synapsin I, laminin receptor, apolipoprotein E and ribosomal proteins S3 and P10 that each promote target cell survival^6, 9^. However, not all EVs released in response to stressors promote neuronal survival. For example, under conditions of LPS-induced stress astrocytes have been shown to shed EVs containing miR-34a that target and reduce translation of the anti-apoptotic protein BCl2, sensitizing neurons to toxic insults^22^.

Stimulation of astrocytes with ATP also induced the release of ADEVs containing modified cargo. ADEV-ATP were enriched with miR-544, miR-532-5p, miR-21, miR-501, miR-628, miR-29a, and miR-99a compared with ADEV-CR. In contrast to the miRNA enriched in ADEV-TNFα and ADEV-IL-1β, miRNA enriched in ADEV-ATP are predicted to promote a neurotrophic and survival response. Several of these miRNAs target key transcriptional regulators, such as Bcl6, the signal transducer and activator of transcription 3, Parkinsonism associated deglycase (MiR-544 (ref. ^68, 69^)), RUNX3 (MiR-532-5p and miR-628 (ref. ^70^)). MiR-501 has been reported to target the lysine 63 specific deubiquitinase CYLD^71^ that deubiquitinates the postsynaptic density protein 95 (PSD95), depleting it from the synapses, disrupting synaptic organization, function and plasticity^72^. Validated targets of miR-21 include the pro-apoptotic protein tissue inhibitor of matrix metalloproteinase-3, and negative regulators of neurite outgrowth the phosphatase and tensin homolog (PTEN), and sprouty homolog 2 (ref. ^73–75^). MiR-29a targets proteins that mediate excitotoxicity, neuronal death and degeneration, including the sterol regulatory element binding protein-1 (SREBP1), p53 upregulated modulator of apoptosis (PUMA) and Neuron Navigator 3 (NAV3). This compilation of enriched miRNAs in ADEV-ATP is consistent with effects on neurons that would promote neuronal survival, strengthen synapses and enhance the connectivity of neural networks. Indeed, we have preliminary data that suggest ADEV-ATP promotes synaptic maturation, and enhances neural network connectivity.

In summary, our data suggest that astrocytes respond to changes in the extracellular microenvironment by modifying the miRNA cargo of EVs. Modifications in ADEV miRNA cargo adjust the activity of target neurons by regulating the translational expression of proteins controlling transcriptional programs essential for synaptic stability and neuronal excitability.

## Materials and methods

### Astrocyte culture

Primary rat cortical astrocyte cell cultures were established and maintained as described previously^76^. Briefly, tissues were isolated from the cerebral cortex of postnatal day 1 Sprague-Dawley rats, cells were mechanically dissociated in Hanks’ balanced salt solution, and plated on poly-d-lysine (Sigma) coated T175 culture flasks (Corning) containing Dulbecco’s modified Eagle’s medium/F-12 media (Gibco BRL) supplemented with 10% fetal bovine serum (Gibco BRL), D-glucose (final concentration 25 mM) (Sigma), and 1% antibiotic/antimitotic solution (104 Unit of penicillin G/ml, 10 mg streptomycin/ml and 25 μg amphotericin B/ml) (Sigma). Type 1 astrocytes were purified by mechanical disassociation of less adherent cells as previously reported^7^. Culture flasks were secured to a rotary shaker (Thermo Scientific) and rotated at 200 r.p.m. in a tissue culture incubator (Thermo Scientific) for 18 h at 37 °C in a 5% CO_2_ environment. Less adherent cells were removed with the media and fresh media added to the cells. Cells were split when close to confluent and at passage 2 cultures were 98% GFAP^+^ astrocytes with type I morphology. Astrocytes were used for experiments between passages 3–10.

### Primary neuron culture

Primary hippocampal and cortical neurons were prepared from day 18 embryos of Sprague-Dawley rats as described previously^77^. In brief, hippocampal tissues were separately dissociated by gentle trituration in a calcium-free Hank’s balanced salt solution and centrifuged at 1000×*g*. Cells were resuspended in Neurobasal media (Gibco) containing B27 supplement (Thermo Fisher Scientific), 1% antibiotic/antimitotic solution (104 Unit of penicillin G/ml, 10 mg streptomycin/ml and 25 μg amphotericin B/ml) (Sigma), and plated at a density of 40,000 cells/ml in 96-well plates (Corning) coated with polyethyleneimine (Sigma) or 160,000 cells/ml in multichannel electrode array chambers (Harvard Apparatus) coated with polyethyleneimine (Sigma) and laminin (Sigma). Three hours after plating the media was replaced with serum-free Neurobasal medium containing 1% B-27 supplement (Gibco). Immunofluorescent staining showed that cultures were >98% MAP-2+ neurons. Hippocampal cultures were used after 3 days of culture in vitro (DIV) as developing neurons, and 14 DIV as mature neurons. All animal procedures were approved by the Johns Hopkins University Animal Care and Use Committee.

### Isolation of ADEVs

Rat primary astrocytes (~80% confluent) were gently washed 3× with warm phosphate-buffered saline (PBS) to remove endogenous EVs. ADEV release was stimulated by the addition of fresh EV-free medium containing ATP (10 μM, Sigma), IL-1β (200 ng/ml, R&D Systems) or TNFα (200 ng/mL, Millipore). Medium was collected 2 h after stimulation, and ADEVs were isolated by a differential ultracentrifugation as described previously^78^. In brief, the collected medium was centrifuged at 2700×*g* for 15 min at 20 °C to remove cellular debris. The supernatant was then ultra-centrifuged at 10,000×*g* for 30 min at 4 °C to remove larger microvesicles and apoptotic bodies. The resulting supernatant was ultra-centrifuged at 100,000×*g* for 3 h at 4 °C to obtain the ADEV pellet. This pellet is washed twice in PBS to ensure removal of non-specific proteins and debris.

### Nanoparticle tracking analysis

Size and number of ADEVs were quantified using a ZetaView Nanoparticle Tracker (Particle Metrix GmBH, Meerbusch, Germany), and corresponding ZetaVeiw software (8.03.04.01). A nanosphere size standard (100 nm diameter; Thermo Scientific) was used to calibrate the instrument prior to readings. Instrument pre-acquisition parameters were set to a temperature of 23 °C, sensitivity of 65, frame rate of 30 frames per second (fps), shutter speed of 100, and a laser pulse duration equal to that of shutter duration. Post-acquisition parameters were set to a minimum brightness of 25, maximum size of 200 pixels, and a minimum size of 10 pixels. For each sample 1 mL of diluted EVs were injected into the sample-carrier cell and the particle count was measured at five positions, with two cycles of reading per position. The cell was washed with PBS after every sample. The mean size and concentration of EVs/mL (±SEM) was calculated from four replicate experiments. The coefficient of variation as determined from a pooled sample was 4.8% for size and 4.4% for concentration of EVs.

### Characterization of hippocampal neurites

Neurons were fixed with 4% paraformaldehyde, and permeabilized with 0.1% Triton X-100 in phosphate-buffered saline (PBS-T) for 20 min at room temperature. Cells were blocked for 1 h with 5% normal goat serum, incubated with an anti-MAP2 antibody (1:500, Sigma), washed three times with PBS-T, and incubated for 2 h with Alexa 595 secondary antibody (1:1000, Life Technologies). Neurons were imaged using a ×20 objective on a Zeiss Axio Observer Z1 microscope equipped with a motorized stage (MBF Biosciences), and an OrcaER CCD camera (Hamamatsu Inc). A 6 × 6 image montage was obtained from each culture and neurite length, dendritic complexity, number of nodes, number of dendrites, ends, and total surface area were measured by Neurolucida imaging software (version 11.11.12; MBF Biosciences) using the Autoneuron tracing mode. For each treatment condition, 15–20 neurons were measured per experiment. The results presented are the average of three individual experiments.

### MicroRNA expression assay

ADEVs isolated from *n* = 8 T175 culture flasks were pooled together to obtain sufficient material for an in depth miR analysis. RNA extraction was performed using a miRNeasy micro kit (Qiagen, Valencia, CA, Catalog #217084), and miRNA expression performed by the Johns Hopkins University deep sequencing and microarray core facility using the nCounter Rat miRNA expression assay (Nanostring technologies Inc., Seattle, WA) as previously described^7^. Briefly, 100 ng of total RNA was used as input and small RNAs were bridged with tagged DNA in annealing buffer (94 °C for 1 min, 65 °C for 2 min, 45 °C for 10 min, and hold at 48 °C). Polyethylene glycol and ligation buffer were added for 5 min at 48 °C, then ligase was added (48 °C for 3 min, 47 °C for 3 min, 46 °C for 3 min, 45 °C for 5 min, 65 °C for 10 min, and hold at 4 °C). To remove excess tags and bridges samples were purified using a ligation cleanup enzyme (37 °C for 120 min, 70 °C for 10 min, and hold at 4 °C). RNA was further prepped for hybridization by adding water (40 μl), heating the samples to 85 °C for 10 min, and then immediately placing them on ice. RNA (5 μl) was hybridized to miRNA Reporter CodeSet master mix (20 μl) at 65 °C for 18 h. Hybridized RNAs were bound to nCounter cartridges and analyzed using an nCounter Digital Analyzer (NanoString). Raw intensity values (counts) obtained from NanoString were normalized to the positive control spike-in RNAs followed by quantile normalization. Fold change in expression for experimental treatments was calculated using miRNA expression levels in constitutively released exosomes as a baseline. “Enriched miRNAs” were defined as miRNAs increased 1.5-fold or greater in ADEVs released in response to each stimulus compared to ADEV-CR.

### qRT-PCR for validation of microRNA expression

TaqMan mature miR assays for rno-let-7f, rno-miR-16-5p, rno-miR-214, rno-miR-100, rno-miR-125a-5p, rno-miR-145, rno-miR-107, rno-miR-628, rno-miR-544, rno-miR-598-5p, rno-miR-532-5p, rno-miR-449c-3p, rno-miR-21, rno-miR-421, and rno-miR-501 (Applied Biosystems) were used to quantify the expression levels of respective miRs according to the manufacturer’s protocol. For microRNA analysis of ADEVs, rno-miR-23a was used as housekeeping control as its expression remained unchanged with the various stimuli. For miRNA analysis of astrocytes U6 small nuclear RNA (snRNA) was used as housekeeping control. Relative expression was calculated as described previously^79^.

### Western blot analysis

Whole-cell lysates were prepared using RIPA buffer (50 mM Tris-HCl, pH 8; 150 mM NaCl; 1% Nonidet P-40; 0.5% sodium deoxycholate; and 0.1% SDS) and protein quantification was carried out using Pierce BCA protein assay (Thermo Scientific). Proteins (5–15 μg loading) were resolved on 10% SDS-polyacrylamide gels and transferred onto nitrocellulose membranes using iBlot (Invitrogen). Membranes were blocked in 5% non-fat dry milk in TBS containing 0.1% Tween-20 and then incubated overnight at 4 °C with primary antibodies directed against NTRK3 (1:1000; Cell Signaling Technology), Bcl2 (1:1000; Cell Signaling Technology) and actin (1:5000; Sigma-Aldrich). Secondary antibodies were HRP-conjugated anti-rabbit IgG (1:5000; Cell Signaling Technology), or HRP-conjugated anti-mouse IgG (1:5000; Cell Signaling Technology) as appropriate for 1 h at room temperature. Blots were developed using Enhanced Chemiluminescent Substrate (Thermo Scientific), imaged and quantified using G:Box imaging system (Syngene).

### 3′-UTR luciferase reporter assay

NTRK3 3′-UTR was amplified from genomic DNA using the following primers: forward primer, 5′-AAATAGCTAGCAAACTCCTTTTAAGCCTC-3′, and reverse primer, 5′- AGCCCTCTAGATTTCATTAGAAAATAAAGTGT-3′. The first 2000 nucleotides of Bcl2 3′-UTR were amplified from genomic DNA using the following primers: forward primer, 5′-AAATAGCTAGCTTTGTGGAACTGTACGGC-3′, and reverse primer, 5′-AGTTCCTCTAGATTTAAAGCAGCTTTCGAA-3′. The PCR products were cloned into pmiRGLO luciferase reporter vector (Promega) between NheI and XbaI restriction sites. The predicted miR-125a-5p binding site in NTRK3 3′-UTR was mutated using the following primers: 5′-CAGGAAGGGCCTTTGTGGCTGATTCCCTTGGTCC-3′ and 5′-GGACCAAGGGAATCAGCCACAAAGGCCCTTCCTG-3′. The predicted miR-16-5p binding site in NTRK3 3′-UTR was mutated using the following primers: 5′-CGAGGTCACTAAGAGGAGTGCATGGTATCCTGGGAG-3′ and 5′-CTCCCAGGATACCATGCACTCCTCTTAGTGACCTCG-3′. The predicted miR-16-5p binding site in Bcl2 3′-UTR was mutated using the following primers: 5′-CCAGCCTCCACGAGCCAGAAAGCCAGCTTCC-3′ and 5′- GGAAGCTGGCTTTCTGGCTCGTGGAGGCTGG-3′. QuikChange site-directed mutagenesis kit (Agilent Technology) was used for site directed mutagenesis.

HEK293T cells were cotransfected with NTRK3-3′-UTR or Bcl2-3′-UTR luciferase reporter vectors and mimics for miR-125a-5p, miR-16-5p or a scrambled microRNA mimic (Sigma), using lipofectamine 3000 transfection reagent (Invitrogen). Luciferase activity was measured at 48 h after transfection using Dual Glo Luciferase Assay Reagent (Promega) according to the manufacturer’s protocol.

### Encapsulation of miR-125a-5p, miR-16-5p, and miR-SC in artificial EVs using DOTAP

N-[1-(2,3-Dioleoyloxy)propyl]-N,N,Ntrimethylammonium methylsulfate (DOTAP) (Sigma) was incubated in HBS buffer (20 mM HEPES, 150 mM NaCl, pH 7.4) for 5 min followed by mixing miR-125a-5p, miR-16-5p, or miR-SC diluted in HBS buffer. DOTAP and miRNAs were incubated at room temperature for 20 min. The ratio of DOTAP to miRNA was 3:1 (3 μl DOTAP to 1 μg RNA). The resulting artificial EVs were characterized by nanoparticle tracking analysis using ZetaView as described in previous sections. Hippocampal neurons (DIV 14) cultured on optically clear bottom 96-well plates were exposed to the artificial EVs at a dose of 50 EVs/neuron. After 48 h neurons were fixed, stained for MAP2, imaged and their dendritic branching parameters were quantified using Neurolucida as described in previous sections.

### Transfection of microRNA inhibitors in ADEVs

Knockdown of miR-125a-5p and miR-16-5p in ADEVs was accomplished using Exo-Fect Exosome Transfection Reagent (Systems Biosciences). Antisense oligonucleotide inhibitors for miR-125a-5p and miR-16-5p (20 pmole; Qiagen) were mixed with Exo-Fect transfection reagent in 150 μl of siRNA buffer with 1 × 10^7^ ADEVs and incubated for 10 min at 37 °C. Transfected exosomes were precipitated and resuspended in 300 μl 1× PBS.

### Neuronal network activity

Primary neurons were grown on multichannel electrode array plates (MultiChannel Systems 60MEA200/30iR-Ti). Recordings were conducted using an MEA2100 system (MultiChannel Systems) on a stage heated to 37 °C. Voltage, spike, and burst measurements were made using MC_RACK software and filtered using a second order butterworth filter with a 200 Hz cutoff frequency. Spikes were identified as instantaneous time points of voltages that exceed a threshold of at least five standard deviations from baseline. Bursts were identified as clusters of at least four spikes occurring within 100 ms of each other. Only electrodes that showed spontaneous spike activity and low baseline noise (<50 µV) were included for analysis. When neurons exhibited spontaneous spike activity (DIV 21–26) they were exposed to ADEVs (50 ADEVs/cell), and activity was measured at 24 h following exposure. Spike and burst rates were normalized to pre-treatment rates to control for differences in baseline activity.

### Statistical analyses

Group differences were determined by ANOVA with Tukey post hoc comparisons unless otherwise specified. Hierarchical clustering was conducted using log 2 values of normalized median counts. Heat maps and clustering were generated using “gplots” (R package version 3.1.1) (https://cran.r-project.org/web/packages/gplots/index.html).

## Electronic supplementary material

miRNA exosome NT3 Supplementary Figure legends and tables

Supplementary Figure 1

Supplementary Figure 2

Supplementary Figure 5

Supplementary Figure 3

Supplementary Figure 4

Supplementary Figure 6

Supplementary Figure 7
