# Supplementary material for: TNFα and IL-1β modify the miRNA cargo of astrocyte shed extracellular vesicles to regulate neurotrophic signaling in neurons
Source: Cell Death Dis. 2018 Mar 5;9(3):363. doi: 10.1038/s41419-018-0369-4 (PMC5838212; doi:10.1038/s41419-018-0369-4)
Supplement: Supplementary file 1 — miRNA exosome NT3 Supplementary Figure legends and tables [file 41419_2018_369_MOESM1_ESM.docx]

**Supplementary Figure 1: ADEV-ATP promotes neurite outgrowth in developing neurons. (a)** Representative fluorescent images of DIV3 MAP2 immunopositive hippocampal neurons (top panels) and Neurolucida dendrite tracings (bottom panels) following a 48h exposure to the indicated concentrations of ADEV-ATP. Quantitative data show **(b)** dendritic complexity, **(c)** surface area, **(d)** dendritic length, **(e)** number of ends, **(f)** nodes, and **(g)** total dendrite number for DIV 3 hippocampal neurons treated with the indicated concentrations of ADEV-ATP for 24h, 48h or 72h. Data are mean ± SEM of 15-20 neurons from 3 independent experiments. One-way ANOVA with Tukey’s posthoc comparisons. * = *p* < 0.05, ** = *p* < 0.01, *** = *p* < 0.001 compared to control.

**Supplementary Figure 2: ADEV-IL-1β reduces neurite outgrowth in developing neurons**. **(a)** Representative fluorescent images of DIV 3 MAP2 immunopositive hippocampal neurons (top panels) and Neurolucida dendrite tracings (bottom panels) following a 48h exposure to the indicated concentrations of ADEV-IL-1β. Quantitative data show **(b)** dendritic complexity, **(c)** surface area, **(d)** dendritic length, **(e)** number of ends, **(f)** nodes, and **(g)** total dendrite number for the indicated concentrations of ADEV-IL-1β. Data are mean ± SEM of 15-20 neurons from 3 independent experiments. One-way ANOVA followed by Tukey’s posthoc comparisons. # = *p* < 0.05, ## = *p* < 0.01, ### = *p* < 0.001 compared to control.

**Supplementary Figure 3: Inhibition of IL-1β and TNFα activity in neurons does not prevent the detrimental effects of ADEV-IL-1β or ADEV-TNFα on dendrite complexity. (a)** Representative fluorescent images of MAP2 immunopositive hippocampal neurons (top panels) and Neurolucida dendrite tracings (bottom panels). Quantitative data show **(b)** dendritic complexity, **(c)** surface area, **(d)** dendritic length, **(e)** number of ends, **(f)** nodes, and **(g)** total dendrite number. Data are mean ± SEM of 15 neurons from 3 independent experiments. One-way ANOVA followed by Tukey’s posthoc comparisons. # = *p* < 0.05, ### = *p* < 0.001 compared to control.

**Supplementary Figure 4: Bioinformatic pathway analysis of mRNAs targeted by miRNAs enriched in ADEV-ATP** Pathway analysis of predicted microRNA targets enriched in ADEV-IL-ATP using DIANA miRPATH. Heat map shows level of significance of enriched pathways. Darker colors (orange to red) indicate more significant enrichment of predicted microRNA targets belonging to a particular pathway.

**Supplementary Figure 5: Artificial EVs containing miR-125a-5p and miR-16-5p reduce neurite outgrowth and dendritic complexity. (a)** Representative fluorescent images of MAP2 immunopositive hippocampal neurons (top panels) and Neurolucida dendrite tracings (bottom panels). Quantitative data show **(b)** dendritic complexity, **(c)** surface area, **(d)** dendritic length, **(e)** number of ends, **(f)** nodes, and **(g)** total dendrite number. Data are mean ± SEM of 15 neurons from 3 independent experiments. One-way ANOVA followed by Tukey’s posthoc comparisons. # = *p* < 0.05, ## = *p* < 0.01, ### = *p* < 0.001 compared to control.

**Supplementary Figure 6: ADEVs secreted in response to IL-1β do not affect neuronal connectivity. (a,d,g,j)** Binned raster plot using for **(a)** control neurons at baseline (0 h), **(d)** control neurons after 24 h, **(g)** neurons before treatment with ADEV-IL-1β and **(j)** neurons 24h after treatment with ADEV-IL-1β. The first 10 network bursts (peaks labeled 1-10) were analyzed for functional connectivity. **(b,e,h,k)** Representative graphs showing spikes (black dots) and bursts (red lines) of the first 5 network bursts. **(c,f,i,l)** Connectivity maps for the first 5 network bursts in hippocampal neurons grown on MEA plates and treated with the stated conditions. Each node represents an electrode on the plate. Color of edges connecting the nodes represent burst correlation between the electrodes. A bin size of 0.01 sec and correlation threshold of 0.5 was used to generate connectivity maps.

**Supplementary Figure 7: ADEV-IL-1β reduces neuronal activity.** Scatter plots from second set of MEA experiments showing quantitation of spike and burst rates for **(a,b)** Control, **(c,d)** ADEV-IL-1β (particle dose of 50 ADEVs/cell), **(e,f)** ADEV-IL-1β + Scrambled oligonucleotide (Scr In, 20 pmol), and **(g,h)** ADEV-IL-1β + oligonucleotide inhibitors for miR-125 and miR-16 (Combined In, 20 pmol each). Data are mean ± SEM. Paired t-tests were performed to compare spike and burst rate of each electrode before and after treatment. *** = *p* < 0.001 increased compared to baseline and ### = *p* < 0.001 decreased compared to baseline.

**Tables**

**Supplementary table 1: Average size and concentration of ADEVs**

| **Stimulus** | **Size (nm)** | **p-value** | **Concentration** | **p-value** |
| --- | --- | --- | --- | --- |
| TFW | 105.93 (±2.7) |  | 6.7(±0.17)X10^9^ |  |
| ATP (10 μM) | 86.03 (±4.9) | 0.004 | 8.8(±0.51)X10^9^ | <0.001 |
| IL-1β (200 ng/mL) | 94.87 (±6.1) | 0.09 | 9.1(±0.23)X10^9^ | <0.001 |
| TNFα (200 ng/mL) | 100.9 (±4.9) | 0.6 | 8.3(±0.56)X10^9^ | 0.006 |

**Supplementary table 2: MicroRNAs enriched in ADEVs secreted in response to** **ATP, IL-1β or TNFα**

| **Fold change compared to constitutively released ADEVs** | | | |
| --- | --- | --- | --- |
| **microRNA** | **ADEV-ATP** | **ADEV-IL-1β** | **ADEV-TNFα** |
| rno-let-7a | 0.88 | 6.78 | 5.99 |
| rno-let-7c | 0.18 | 1.49 | 0.48 |
| rno-let-7d | 0.99 | 1.53 | 1.14 |
| rno-let-7f | 0.44 | 4.19 | 1.05 |
| rno-miR-100 | 0.58 | 2.05 | 0.34 |
| rno-miR-107 | 1.02 | 1.67 | 3.12 |
| rno-miR-1224 | 0.73 | 1.27 | 1.81 |
| rno-miR-125a-5p | 1.00 | 1.94 | 1.85 |
| rno-miR-125b-5p | 1.58 | 1.58 | 1.45 |
| rno-miR-145 | 1.42 | 1.35 | 3.19 |
| rno-miR-16 | 0.15 | 2.76 | 2.11 |
| rno-miR-199a-3p | 0.62 | 1.15 | 1.55 |
| rno-miR-208 | NA | 0.65 | 2.11 |
| rno-miR-21 | 2.11 | 0.54 | 1.07 |
| rno-miR-214 | 0.78 | 2.76 | 1.79 |
| rno-miR-24 | 0.99 | 1.70 | 1.75 |
| rno-miR-27b | 0.97 | 0.22 | 6.63 |
| rno-miR-29a | 1.68 | 4.92 | 5.56 |
| rno-miR-501 | 2.02 | 0.52 | 1.55 |
| rno-miR-532-5p | 2.36 | 0.40 | 1.56 |
| rno-miR-544 | 4.18 | 0.90 | 2.58 |
| rno-miR-598-5p | 2.80 | 0.79 | 2.26 |
| rno-miR-628 | 1.76 | 0.28 | 2.97 |
| rno-miR-99a | 1.65 | 1.98 | 1.36 |

**Supplementary table 3: Relative expression level of microRNAs detected in ADEVs released in response to respective stimuli**

|  | **Relative Expression Level** | | | |
| --- | --- | --- | --- | --- |
| **microRNA** | **ADEV-CR** | **ADEV-ATP** | **ADEV-IL-1β** | **ADEV-TNFα** |
| rno-let-7a | 49.475 | 43.295 | 335.316 | 296.233 |
| rno-let-7b | 1231.496 | 1455.356 | 1457.122 | 725.540 |
| rno-let-7c | 1745.083 | 322.556 | 2591.451 | 842.480 |
| rno-let-7d | 898.514 | 893.461 | 1375.889 | 1028.146 |
| rno-let-7e | 581.790 | 67.058 | 513.905 | 142.404 |
| rno-let-7f | 174.512 | 77.548 | 730.678 | 182.913 |
| rno-let-7i | 604.810 | 56.055 | 585.302 | 260.699 |
| rno-miR-1 | 19.485 | NA | 27.156 | NA |
| rno-miR-100 | 447.922 | 260.988 | 918.976 | 153.414 |
| rno-miR-106b | 129.668 | NA | 43.927 | NA |
| rno-miR-107 | 283.210 | 287.493 | 472.542 | 884.940 |
| rno-miR-1224 | 204.600 | 149.309 | 259.878 | 369.983 |
| rno-miR-125a-5p | 554.385 | 551.802 | 1074.622 | 1022.932 |
| rno-miR-125b-5p | 2412.483 | 3823.200 | 3823.200 | 3506.143 |
| rno-miR-126 | 75.644 | NA | 70.942 | 66.346 |
| rno-miR-130a | 1489.893 | 160.475 | 788.037 | 597.882 |
| rno-miR-132 | 137.844 | 33.246 | 158.700 | 30.338 |
| rno-miR-135a | 425.049 | NA | 279.363 | NA |
| rno-miR-142-3p | 92.873 | NA | 69.378 | 19.369 |
| rno-miR-143 | 51.176 | NA | 41.144 | NA |
| rno-miR-144 | 164.400 | NA | 62.037 | 73.654 |
| rno-miR-145 | 858.093 | 1222.739 | 1155.241 | 2740.159 |
| rno-miR-148b-3p | 85.759 | NA | 51.564 | NA |
| rno-miR-151 | 80.829 | 26.278 | 209.914 | 51.996 |
| rno-miR-152 | 458.063 | 16.013 | 359.902 | 104.940 |
| rno-miR-154 | 157.010 | 16.013 | 96.553 | 28.201 |
| rno-miR-15b | 319.834 | 33.246 | 422.593 | 182.913 |
| rno-miR-16 | 677.341 | 104.202 | 1872.461 | 1426.862 |
| rno-miR-17-5p | 33.702 | NA | 37.783 | NA |
| rno-miR-181a | 1457.122 | 1371.181 | 566.985 | 551.802 |
| rno-miR-188 | 82.702 | NA | 72.819 | NA |
| rno-miR-190b | 30.498 | 27.916 | 25.163 | 30.338 |
| rno-miR-191 | 108.766 | 18.184 | 245.783 | 60.224 |
| rno-miR-1949 | 306.605 | NA | 193.637 | NA |
| rno-miR-195 | 47.590 | NA | 37.783 | NA |
| rno-miR-199a-3p | 728.694 | 453.159 | 839.164 | 1132.668 |
| rno-miR-199a-5p | 233.200 | 85.283 | 447.320 | 412.151 |
| rno-miR-19a | 42.444 | NA | 18.356 | NA |
| rno-miR-203 | 41.039 | NA | 32.789 | 19.369 |
| rno-miR-204 | 239.366 | NA | 270.556 | 80.742 |
| rno-miR-206 | 33.173 | 21.638 | 37.200 | 60.224 |
| rno-miR-208 | 224.517 | NA | 145.688 | 473.891 |
| rno-miR-20a+20b-5p | 118.732 | 53.090 | 105.675 | 66.346 |
| rno-miR-21 | 1631.405 | 3160.475 | 873.969 | 1749.790 |
| rno-miR-214 | 64.498 | 50.606 | 178.149 | 115.647 |
| rno-miR-218a | 294.273 | NA | 118.142 | 35.567 |
| rno-miR-22 | 1884.194 | 1879.552 | 898.514 | 2114.205 |
| rno-miR-221 | 37.805 | NA | 48.417 | NA |
| rno-miR-222 | 41.039 | 13.503 | 32.789 | 41.287 |
| rno-miR-23a | 2160.263 | 1792.691 | 2380.622 | 2114.205 |
| rno-miR-23b | 75.644 | NA | 85.356 | 33.221 |
| rno-miR-24 | 428.633 | 424.571 | 728.694 | 442.631 |
| rno-miR-25 | 472.166 | 114.319 | 315.756 | 91.217 |
| rno-miR-26b | 78.790 | NA | 79.281 | 35.567 |
| rno-miR-27a | 626.062 | 395.663 | 557.471 | 785.287 |
| rno-miR-27b | 187.617 | 181.313 | 41.518 | 1244.487 |
| rno-miR-28 | 53.605 | 18.184 | 114.283 | 49.229 |
| rno-miR-290 | 1324.039 | NA | 457.892 | 941.819 |
| rno-miR-293 | 32.107 | NA | NA | 49.229 |
| rno-miR-29a | 290.579 | 488.472 | 1430.414 | 1614.773 |
| rno-miR-29b | 949.302 | 33.246 | 302.671 | 346.944 |
| rno-miR-29c | 343.498 | 39.612 | 137.647 | 91.217 |
| rno-miR-301a | 90.478 | NA | 83.175 | 46.342 |
| rno-miR-30a | 392.639 | NA | 142.167 | NA |
| rno-miR-30b-5p | 70.888 | NA | 81.146 | NA |
| rno-miR-30c | 401.942 | 129.445 | 613.449 | 236.478 |
| rno-miR-30d | 143.224 | 36.275 | 231.544 | 64.035 |
| rno-miR-32 | 47.590 | 72.823 | 46.578 | 83.153 |
| rno-miR-322 | 69.390 | NA | 109.366 | 37.513 |
| rno-miR-324-5p | 16.610 | NA | 57.773 | NA |
| rno-miR-325-3p | 115.965 | 114.319 | 142.905 | 98.602 |
| rno-miR-328a | 145.478 | NA | 82.244 | NA |
| rno-miR-335 | 15.690 | 12.071 | 12.071 | 19.369 |
| rno-miR-344a-3p | 49.829 | 54.677 | 63.237 | 83.153 |
| rno-miR-345-5p | 108.766 | NA | 76.007 | NA |
| rno-miR-34a | 16.610 | NA | 37.117 | NA |
| rno-miR-34c | 19.485 | NA | 67.705 | 37.513 |
| rno-miR-3563-5p | 39.175 | 19.904 | 30.310 | 19.369 |
| rno-miR-3566 | 14.246 | 24.917 | 17.787 | NA |
| rno-miR-3567 | 17.787 | 14.271 | 22.283 | NA |
| rno-miR-3569 | 20.183 | 30.028 | 35.183 | NA |
| rno-miR-3571 | 111.512 | 114.319 | 106.747 | 126.637 |
| rno-miR-3577 | 29.356 | 38.073 | 68.735 | NA |
| rno-miR-3578 | 31.030 | 46.245 | 49.475 | 51.996 |
| rno-miR-3579 | 22.947 | 27.009 | 22.283 | NA |
| rno-miR-3581 | 33.173 | 48.147 | 48.267 | 41.287 |
| rno-miR-3583-3p | 457.195 | 719.770 | 821.043 | 457.276 |
| rno-miR-3585-3p | 39.175 | 23.237 | 23.396 | 41.287 |
| rno-miR-3585-5p | 34.637 | 30.028 | 33.475 | 46.342 |
| rno-miR-3586-5p | 39.175 | 30.028 | 31.030 | NA |
| rno-miR-3589 | 86.491 | 139.174 | 111.512 | 70.199 |
| rno-miR-3590-3p | 47.213 | 61.217 | 71.641 | 77.126 |
| rno-miR-3590-5p | 83.781 | 97.263 | 90.977 | 77.126 |
| rno-miR-3592 | 24.421 | 24.917 | 39.175 | 43.110 |
| rno-miR-3593-5p | 90.977 | 89.026 | 83.781 | 95.346 |
| rno-miR-3594-3p | 264.046 | 273.116 | 493.164 | 217.887 |
| rno-miR-3594-5p | 152.408 | 246.477 | 250.650 | 146.441 |
| rno-miR-3595 | 70.476 | 67.058 | 71.641 | 62.363 |
| rno-miR-3596b | 29.356 | 41.185 | 46.445 | NA |
| rno-miR-3596c | 60.104 | 97.263 | 86.491 | 115.647 |
| rno-miR-3596d | 22.947 | 16.013 | 16.765 | NA |
| rno-miR-361 | 97.805 | NA | 66.007 | NA |
| rno-miR-365 | 188.824 | 20.640 | 148.800 | 33.221 |
| rno-miR-378 | 61.732 | NA | 33.498 | 19.369 |
| rno-miR-381 | 17.787 | 18.184 | 15.978 | NA |
| rno-miR-411 | 266.234 | NA | 122.264 | 51.996 |
| rno-miR-421 | 98.447 | 198.763 | 155.320 | 133.483 |
| rno-miR-448 | 34.637 | 43.295 | 43.449 | 33.221 |
| rno-miR-449c-3p | 79.075 | 181.313 | 179.618 | 133.483 |
| rno-miR-450a | 273.961 | 67.058 | 165.854 | 277.115 |
| rno-miR-451 | 210.717 | NA | 142.167 | 83.153 |
| rno-miR-455 | 179.618 | 228.785 | 264.046 | 142.404 |
| rno-miR-465 | 62.542 | 72.823 | 61.323 | 37.513 |
| rno-miR-466b | 55.283 | 58.415 | 54.230 | 70.199 |
| rno-miR-466c | 16.765 | 22.370 | 387.375 | 2553.623 |
| rno-miR-466d | 363.473 | 82.730 | 124.970 | 115.647 |
| rno-miR-489 | 52.287 | 80.355 | 75.882 | 64.035 |
| rno-miR-501 | 105.328 | 212.246 | 54.753 | 163.183 |
| rno-miR-511 | 37.322 | NA | NA | 43.110 |
| rno-miR-532-5p | 250.650 | 590.423 | 100.010 | 207.035 |
| rno-miR-539 | 34.637 | 33.246 | 31.030 | 30.338 |
| rno-miR-540 | 26.848 | 23.237 | 29.356 | 28.201 |
| rno-miR-543 | 82.059 | 45.051 | 132.047 | 109.402 |
| rno-miR-544 | 123.405 | 515.545 | 110.973 | 318.685 |
| rno-miR-598-5p | 115.351 | 322.556 | 91.583 | 260.699 |
| rno-miR-628 | 335.316 | 590.423 | 94.000 | 996.233 |
| rno-miR-741-3p | 34.829 | NA | 25.955 | 33.221 |
| rno-miR-7a | 36.210 | NA | 18.356 | 35.567 |
| rno-miR-98 | 147.732 | 48.147 | 209.914 | 37.513 |
| rno-miR-99a | 493.164 | 814.016 | 976.363 | 668.866 |
| rno-miR-99b | 158.233 | 104.202 | 338.083 | 57.186 |
