# Supplementary figures and images for: TNFα and IL-1β modify the miRNA cargo of astrocyte shed extracellular vesicles to regulate neurotrophic signaling in neurons
Source: Cell Death Dis. 2018 Mar 5;9(3):363. doi: 10.1038/s41419-018-0369-4 (PMC5838212; doi:10.1038/s41419-018-0369-4)

Supplementary Figure 1

a

ADEV-ATP (ADEVs/cell)

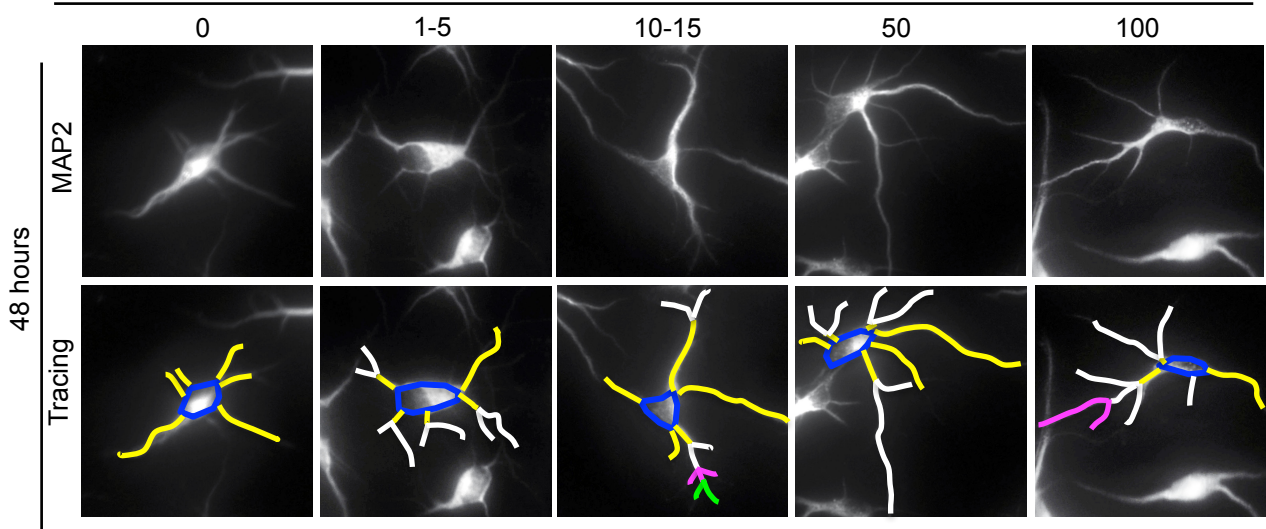

b

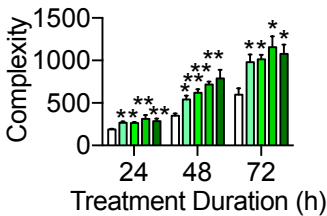

c

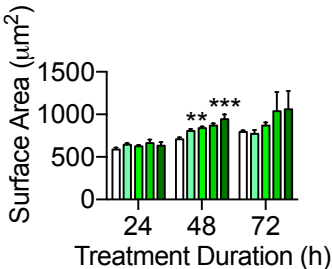

d

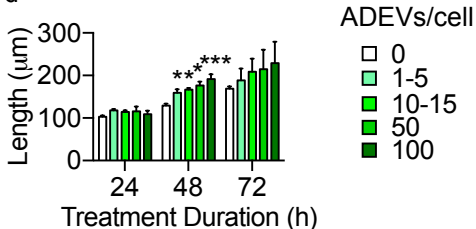

e

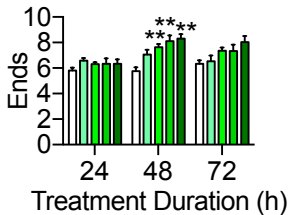

f

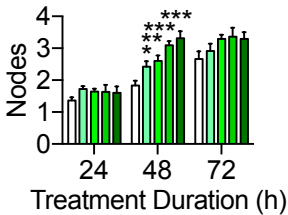

g

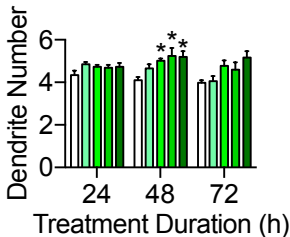

Supplement: Supplementary file 2 — Supplementary Figure 1 [file 41419_2018_369_MOESM2_ESM.pdf]

# Supplementary Figure 2

a

ADEV-IL-1 $\beta$  (ADEVs/cell)

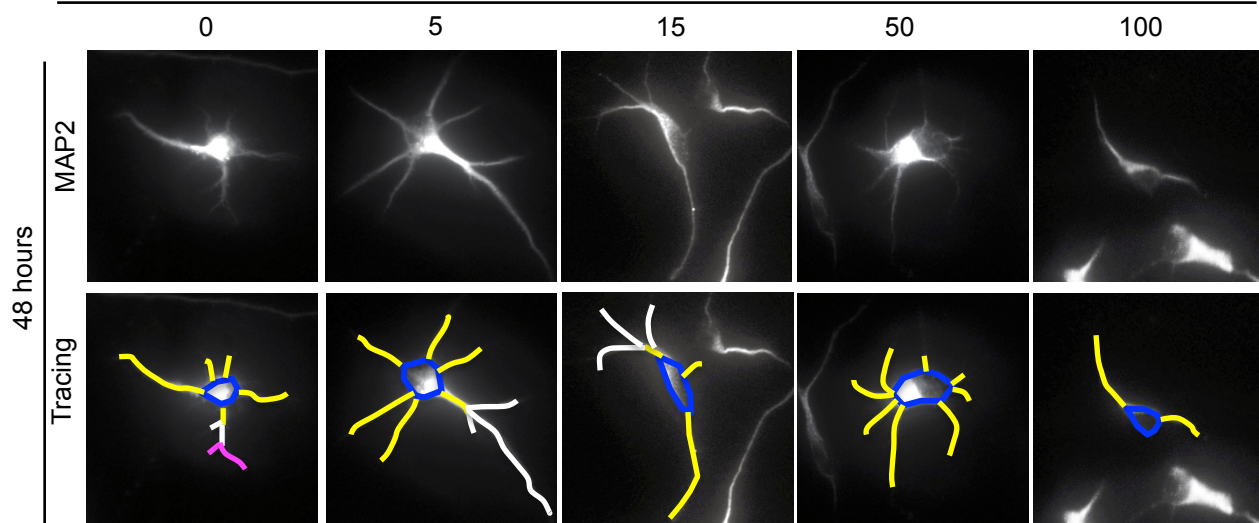

b

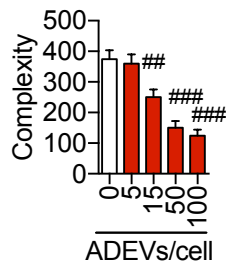

c

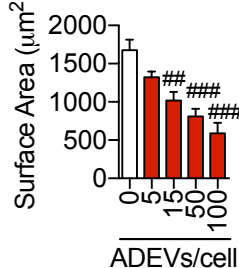

d

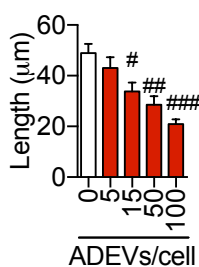

e

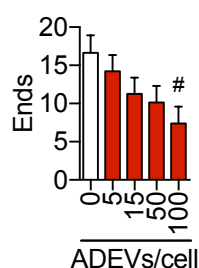

f

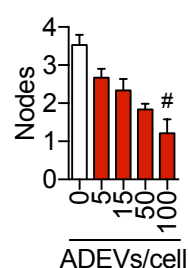

g

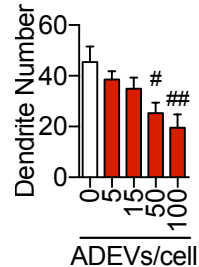

Supplement: Supplementary file 3 — Supplementary Figure 2 [file 41419_2018_369_MOESM3_ESM.pdf]

Supplementary Figure 5

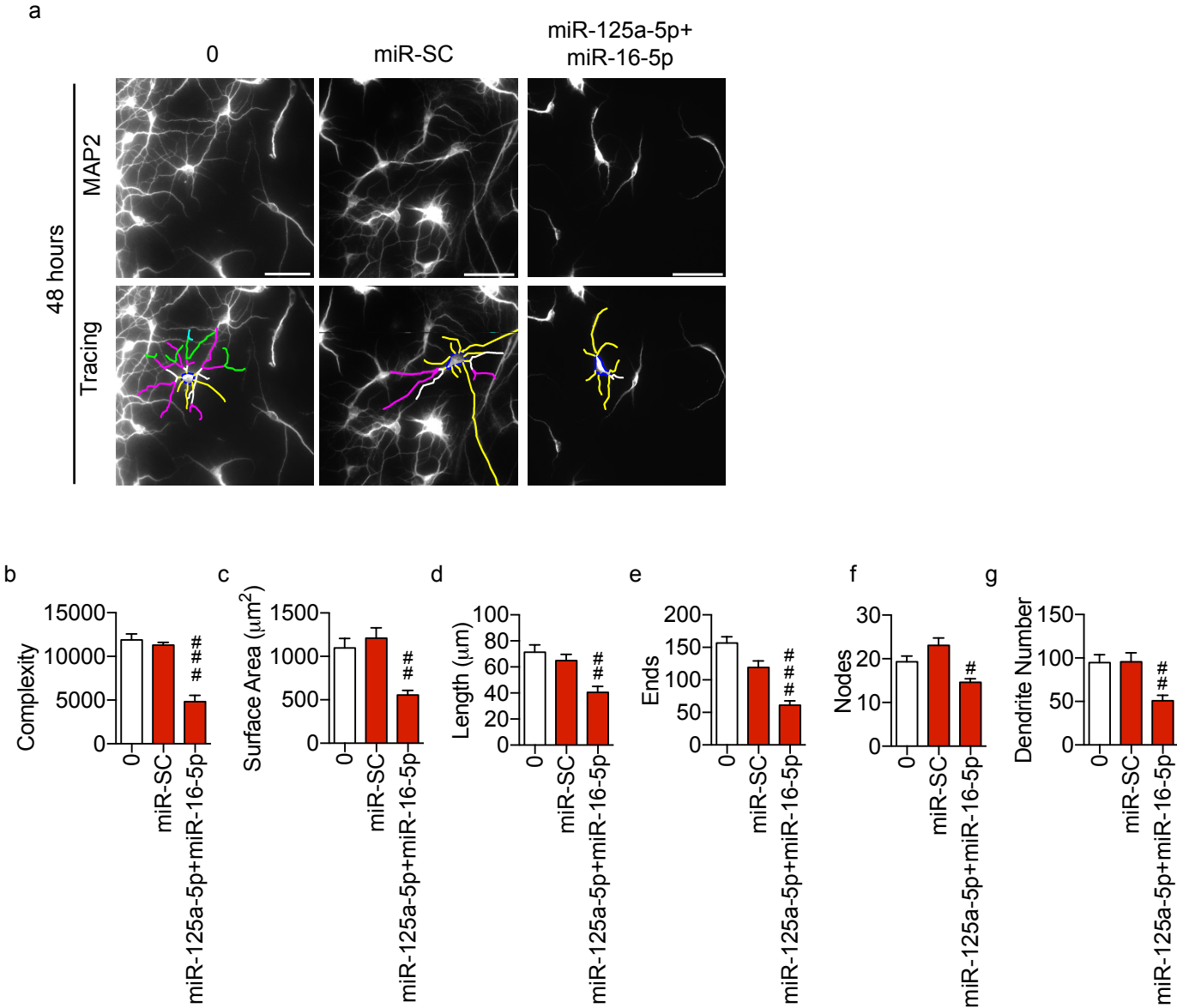

Supplement: Supplementary file 4 — Supplementary Figure 5 [file 41419_2018_369_MOESM4_ESM.pdf]

Supplementary Figure 3

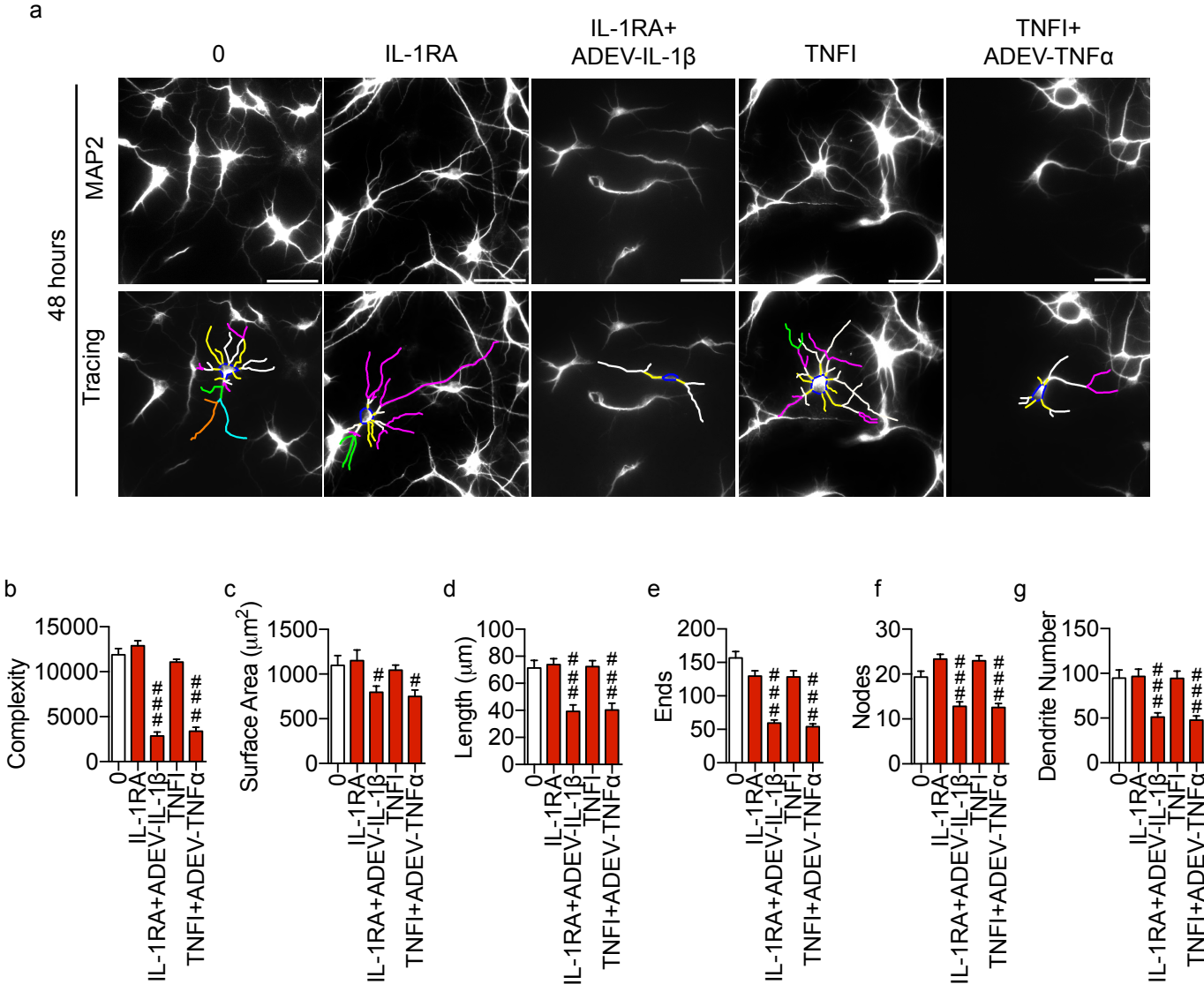

Supplement: Supplementary file 5 — Supplementary Figure 3 [file 41419_2018_369_MOESM5_ESM.pdf]

## Supplementary Figure 4

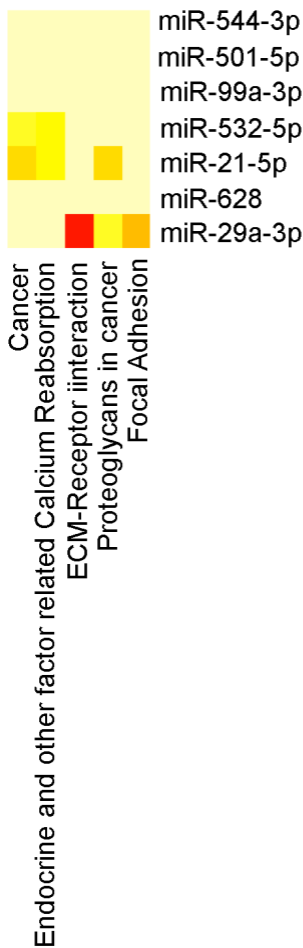

Supplement: Supplementary file 6 — Supplementary Figure 4 [file 41419_2018_369_MOESM6_ESM.pdf]

Supplementary Figure 6

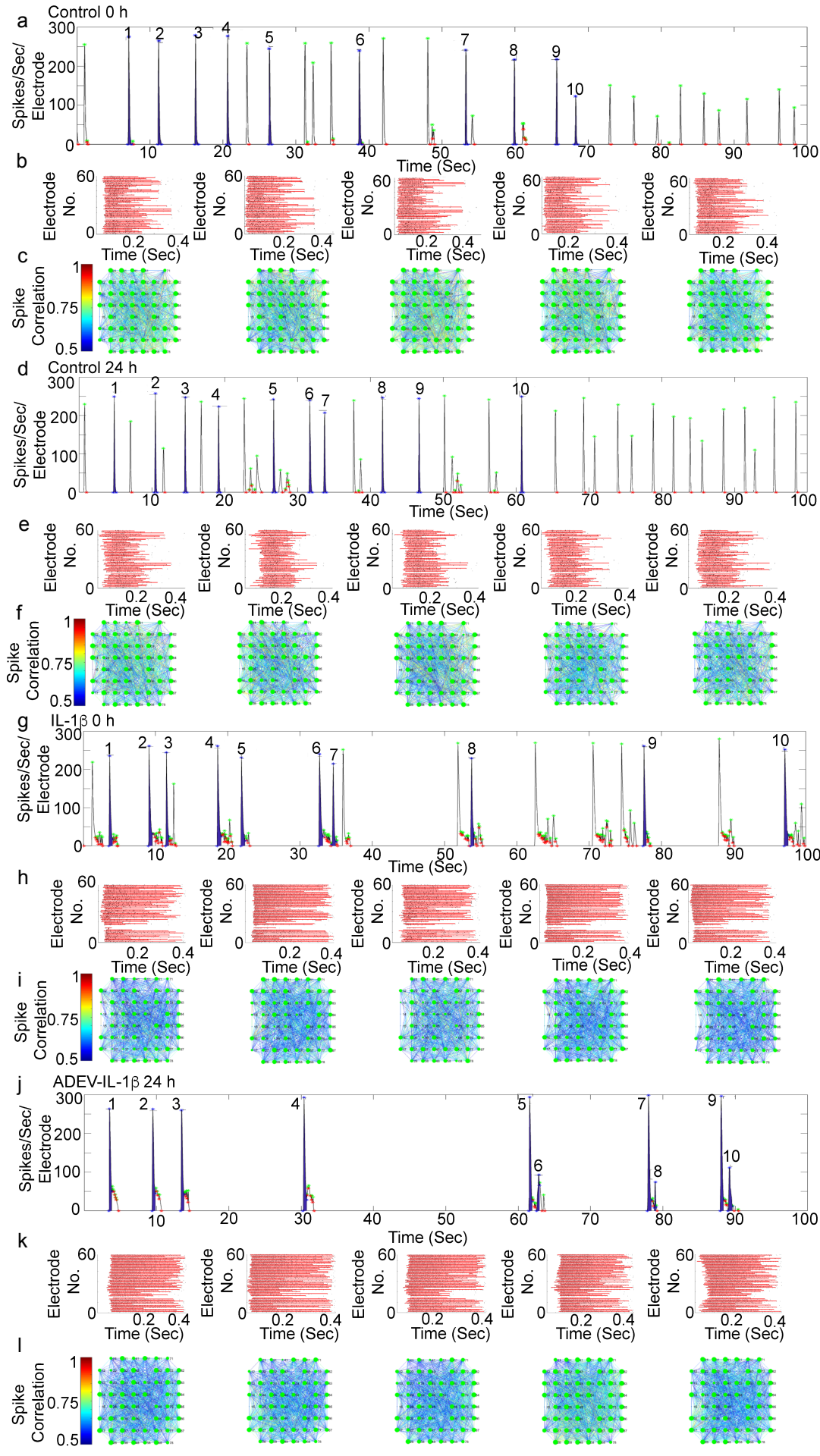

Supplement: Supplementary file 7 — Supplementary Figure 6 [file 41419_2018_369_MOESM7_ESM.pdf]

Supplementary Figure 7

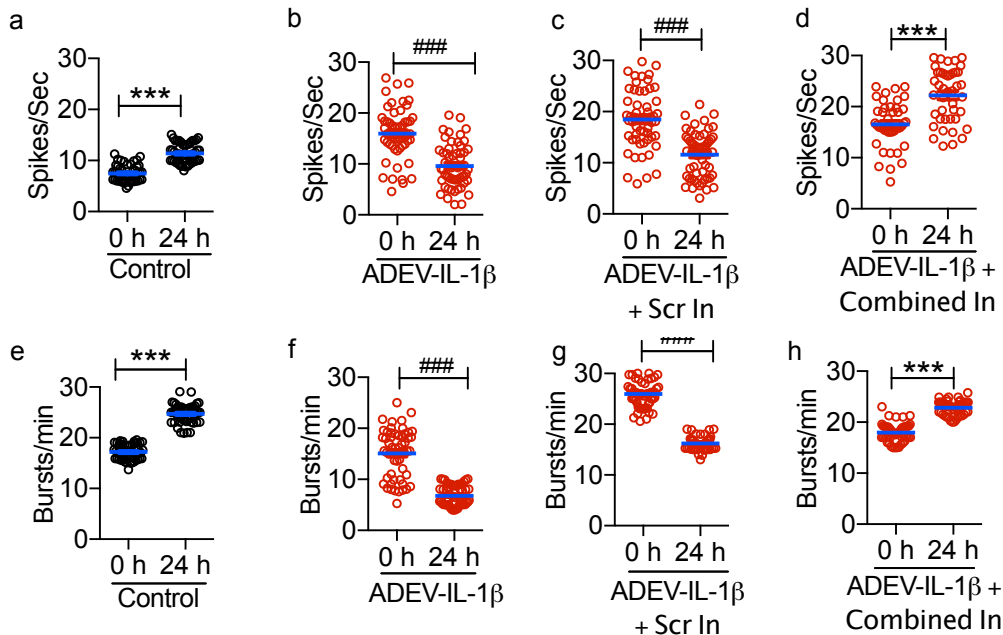

Supplement: Supplementary file 8 — Supplementary Figure 7 [file 41419_2018_369_MOESM8_ESM.pdf]
